# Synthesis and crystal structures of 5,17-di­bromo-26,28-dihy­droxy-25,27-dipropynyloxycalix[4]arene, 5,17-di­bromo-26,28-dipropoxy-25,27-dipropynyloxycalix[4]arene and 25,27-bis­(2-azido­eth­oxy)-5,17-di­bromo-26,28-di­hydroxy­calix[4]arene

**DOI:** 10.1107/S2056989024003785

**Published:** 2024-05-03

**Authors:** Alexander Gorbunov, Stanislav Bezzubov, Maria Malakhova, Vladimir Kovalev, Ivan Vatsouro

**Affiliations:** a Department of Chemistry, Lomonosov Moscow State University, Lenin’s Hills, 1-3, Moscow, 119991, Russian Federation; b N. S. Kurnakov Institute of General and Inorganic Chemistry, Russian Academy of Sciences, Leninskii pr. 31, Moscow 119991, Russian Federation; Vienna University of Technology, Austria

**Keywords:** crystal structure, macrocycles, calix[4]arene, synthesis, NMR study

## Abstract

The calixarenes, 5,17-di­bromo-26,28-dihy­droxy-25,27-dipropynyloxycalix[4]arene (C_34_H_26_Br_2_O_4_, **1**), 5,17-di­bromo-26,28-dipropoxy-25,27-dipropynyloxycalix[4]arene (C_40_H_38_Br_2_O_4_, **2**) and 25,27-bis­(2-azido­eth­oxy)-5,17-di­bromo-26,28-di­hydroxy­calix[4]arene (C_32_H_28_Br_2_N_6_O_4_, **3**) possess a pinched cone mol­ecular shape for **1** and **3**, and a 1,3-alternate shape for compound **2**. In calixarenes **1** and **3**, the cone conformations are additionally stabilized by intra­molecular O—H⋯O hydrogen bonds, while in calixarene **2** intra­molecular Br⋯Br inter­actions consolidate the 1,3-alternate mol­ecular conformation. The dense crystal packing of the cone dialkyne **1** is a consequence of π–π, C—H⋯π and C—H⋯O inter­actions. In the crystal of the diazide **3**, there are large channels extending parallel to the *c* axis, which are filled by highly disordered CH_2_Cl_2_ solvent mol­ecules. Their contribution to the intensity data was removed by the SQUEEZE procedure that showed an accessible void volume of 585 Å^3^ where there is room for 4.5 CH_2_Cl_2_ solvent mol­ecules per unit cell. Rigid mol­ecules of the 1,3-alternate calixarene **2** form a columnar head-to-tail packing parallel to [010] *via* van der Waals inter­actions, and the resulting columns are held together by weak C—H⋯π contacts.

## Abstract 2

The title compounds are two calix[4]arenes with a pinched cone conformation and one with a 1,3-alternate conformation.

## 1. Chemical context

Calixarene macrocycles offer the possibility to combine several functional groups of a different nature and to preorganize them spatially. The polyfunctional nature of calixarenes allows their use in the development of new materials, drugs, substances for medical applications and in other areas of organic chemistry, biochemistry or materials science where supra­molecular organizations are of importance. The versa­tility of calixarenes as mol­ecular platforms is due to the availability of the polyphenolic macrocycles themselves, and to well-developed approaches for the exhaustive and partial modification of phenolic hydroxyl groups and/or aromatic *para*-positions (Asfari *et al.*, 2001 ▸; Vicens *et al.*, 2007 ▸; Bohmer, 2003 ▸; Neri *et al.*, 2016 ▸). The modification of calixarene macrocycles by azide or alkyne functional groups makes them suitable for copper(I)-catalyzed azide-alkyne cyclo­addition (CuAAC) (Song *et al.*, 2014 ▸). Under the usual CuAAC conditions, bis­calixarene (Gorbunov *et al.*, 2021 ▸) or tris­calixarene (Malakhova *et al.*, 2022*a* ▸) mol­ecular semitubes were synthesized, and the processes of intra­molecular oscillations of Ag^+^ inside them were studied (Malakhova *et al.*, 2022*b* ▸). It is expected that grafting of additional substituents into the *para*-positions of phenolic fragments of the azide/alkyne-containing calix[4]arenes, on the one hand, should improve shielding of the inter­nal cavity of the calixarene semitube and, on the other hand, may provide possibilities for further modifications of the multicalixarene assemblies. In this context, we synthesized the *para*-di­bromo-substituted calix[4]arenes **1**–**3** bearing 2-azido­ethyl and propargyl functionalities. The compositions and structures of the synthesized compounds were analyzed by ^1^H, ^13^C NMR (Scheme S1, Figs. S1–S6 in the supporting information), and single-crystal X-ray diffraction. 
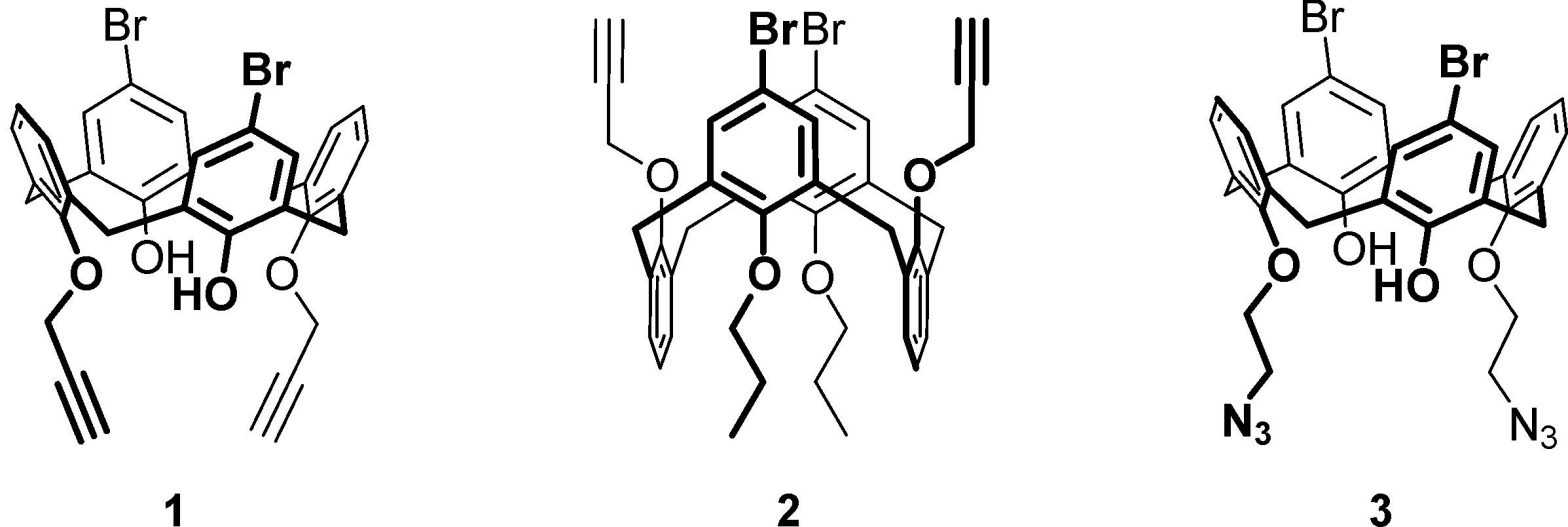


## 2. Structural commentary

**Figure 1:**
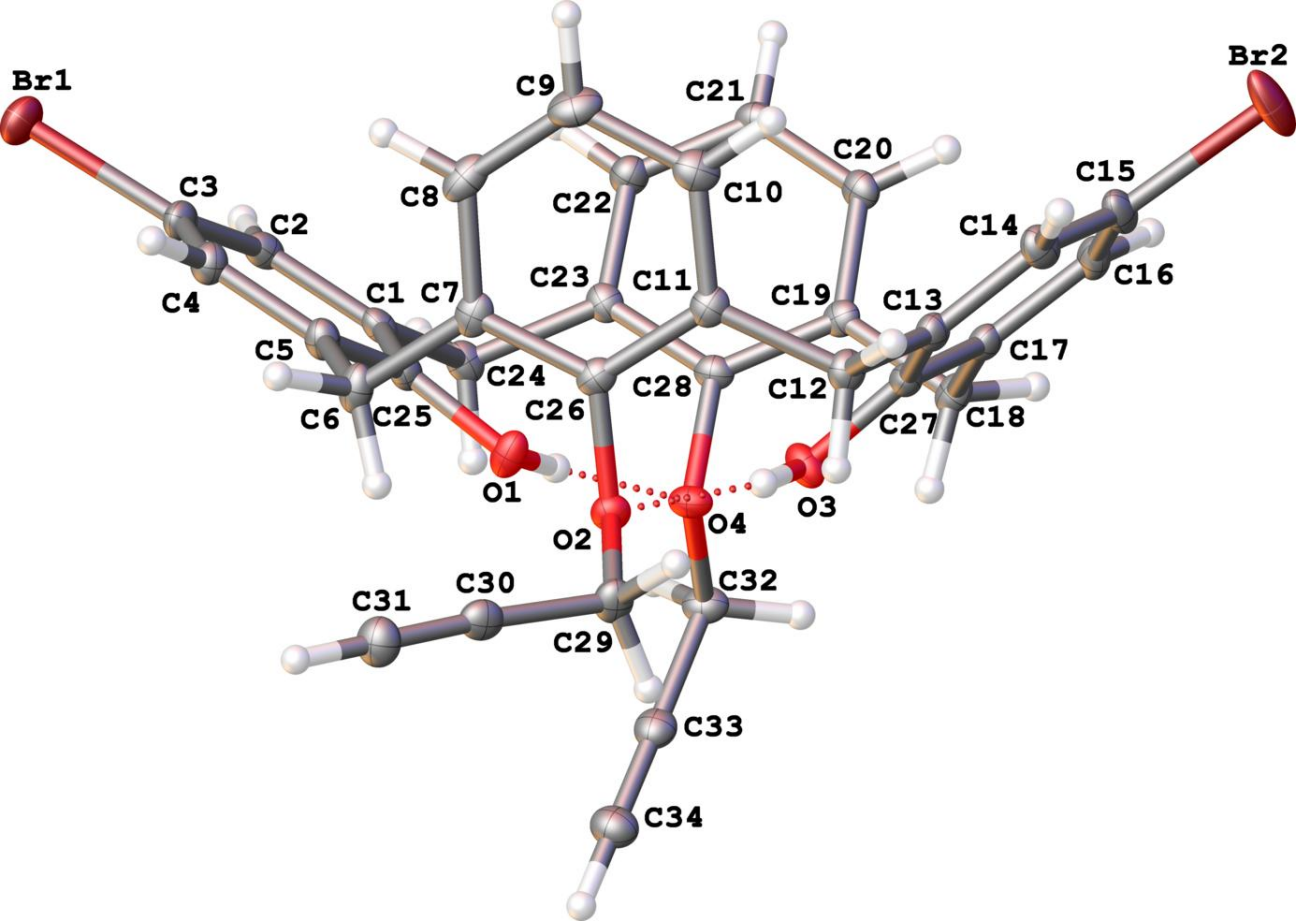
Mol­ecular structure of 5,17-di­bromo-26,28-dihy­droxy-25,27-dipropynyloxycalix[4]arene (**1**), with displacement ellipsoids drawn at the 50% probability level. The minor part of the disordered bromine atom is omitted for clarity. O—H⋯O hydrogen bonds are shown by dotted lines.

**Figure 3:**
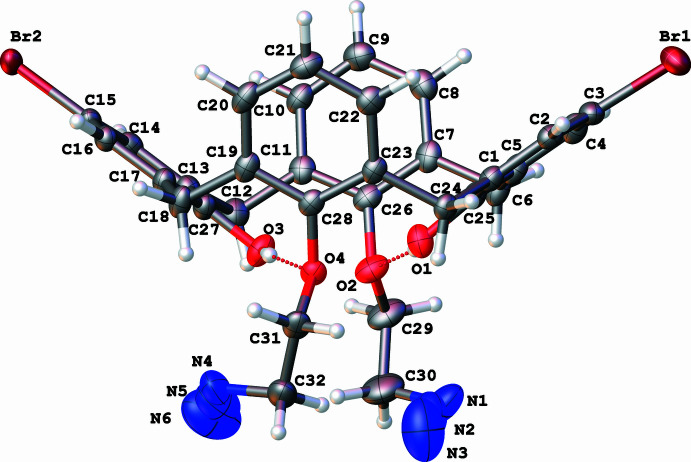
Mol­ecular structure of 25,27-bis­(2-azido­eth­oxy)-5,17-di­bromo-26,28-di­hydroxy­calix[4]arene (**3**), with displacement ellipsoids drawn at the 50% probability level. O—H⋯O hydrogen bonds are shown by dotted lines.

The calix[4]arenes **1** and **3** occupy general positions, while the macrocycle **2** possesses mol­ecular *C*2 symmetry with the twofold rotation axis passing through the center of the calixarene cavity (Figs. 1 ▸–3 ▸ ▸). The cone conformation of **1** is stabilized by moderate intra­molecular O—H⋯O hydrogen bonds (Table 1 ▸, Fig. 1 ▸). The *para*-bromo-substituted rings (the second Br atom (Br’) is generated by the symmetry operation 1 − *x*, *y*, 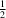 − *z*) are located further apart [*d*(C1–C5/C25_centroid_–C13–C17/C27_centroid_) = 7.4083 (11) Å, inter­planar angle 71.29 (6)°] than the unsubstituted ones [*d*(C7–C11/C26_centroid_–C19–C23/C28_centroid_) = 6.1827 (11) Å, inter­planar angle 34.54 (6)°]. Compound **3** also has a cone conformation supported by intra­molecular O—H⋯O hydrogen bonds (Table 2 ▸, Fig. 3 ▸) with the analogous mutual arrangement of the substituted [*d*(C1–C5/C25_centroid_–C13–C17/C27_centroid_) = 7.4401 (16) Å, inter­planar angle 70.96 (9)°] and unsubstituted rings [*d*(C7–C11/C26_centroid_–C19–C23/C28_centroid_) = 6.0604 (15) Å, inter­planar angle 31.57 (9)°]. Compound **2** possesses a 1,3-alternate conformation with an intra­molecular halogen⋯halogen inter­action [*d*(Br⋯Br’) = 3.9765 (4) Å]. The closer contacts of the bromine atoms leads to a significant increase in the angle between the planes of the corresponding rings [C1–C6, 22.48 (6)°] and, as a result, an almost equal increase of the inter­planar angle between the pair of unsubstituted rings [C8–C13, 21.63 (6)°].

^1^H NMR spectra of compounds **1** and **3** are quite similar and simple due to the highly symmetrical structure of the calixarenes. Indeed, in each spectrum, a singlet corresponding to phenolic hydroxyl groups and two multiplets and a singlet from the aromatic calixarene H atoms are located in the low-field part of the spectrum, while the doublet and triplet from the propargyl groups (for calixarene **1**) and two multiplets from the azido­ethyl fragments (for calixarene **3**) as well as two doublets from the calixarene methyl­ene bridges appear in the middle part of the spectrum. In the ^13^C NMR spectra of both compounds **1** and **3**, the characteristic signal from the methyl­ene bridges at ∼31 ppm reflects a cone shape of the macrocycle. In the case of calixarene **2**, the doublets from the methyl­ene bridges in the ^1^H NMR spectrum appear to be located closer to each other and have an increased spin-spin coupling constant value. In the ^13^C NMR spectrum of **2** the signal of the methyl­ene groups appears downfield shifted with respect to the above cone calixarenes (∼37 ppm), which confirms a 1,3-alternate shape of the macrocycle.

## 3. Supra­molecular features

**Figure 4:**
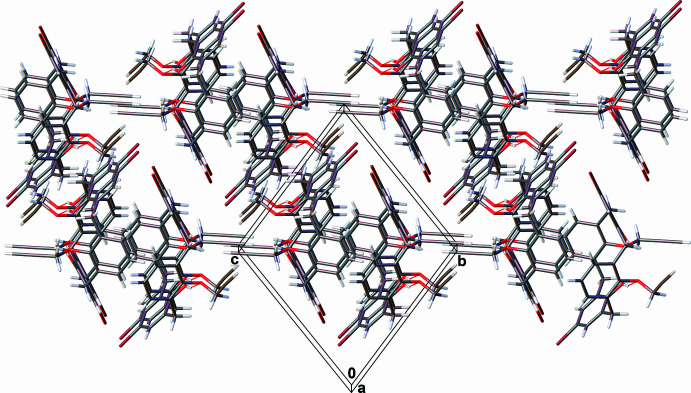
Fragment of the crystal packing of 5,17-di­bromo-26,28-dihy­droxy-25,27-dipropynyloxycalix[4]arene (**1**). The minor part of the disordered bromine atom is omitted for clarity.

In the crystal structure of **1** (Fig. 4 ▸), there are π–π-bonded centrosymmetric dimers [*d*(C21⋯C19-C23/C28_centroid_) = 3.361 (2) Å, centroid-to-centroid shift of 1.862 (3) Å], which are additionally stabilized by C—H⋯π inter­actions between the H20 atom and the centroid of the C1–C5/C25 ring [3.1375 (8) Å, 147.00 (12)°], between the H21 atom and the centroid of the C7–C11/C26 ring [3.0179 (8) Å, 127.00 (12)°] and between the H22 atom and the centroid of the C13–C17/C27 ring [2.7990 (8) Å, 157.54 (12)°]. These dimers are linked into chains extending parallel to [0 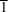 1] *via* C—H⋯O contacts involving the H34 and the O1 atoms [*d*(H⋯O) = 2.3436 (13) Å, C—H⋯O angle = 164.45 (13)°]. The resulting chains are assembled by further C—H⋯π inter­actions between the H31 atom and the mid-point of the triple C33–C34 bond [2.7043 (6) Å, 146.08 (13)°], forming thick layers parallel to (110). These layers are related to each other by inversion centers and are joint by π–π inter­actions [*d*(C27⋯C13-C17/C27_centroid_) = 3.623 (2) Å, centroid-to-centroid shift of 2.276 (3) Å].

**Figure 5:**
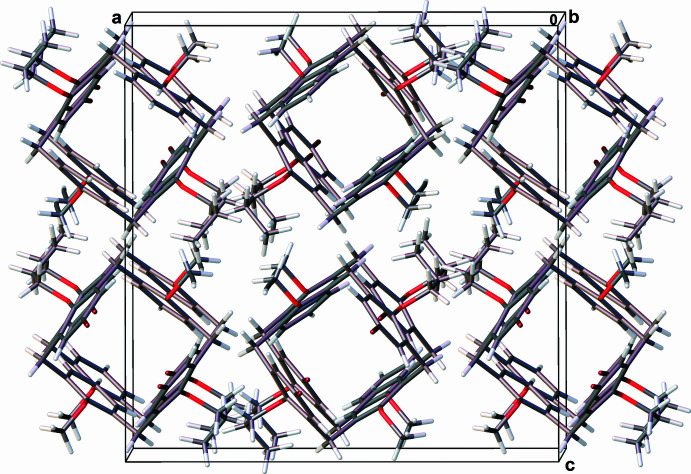
Fragment of the crystal packing of 5,17-di­bromo-26,28-dipropoxy-25,27-dipropynyloxycalix[4]arene (**2**).

In the crystal structure of **2** (Fig. 5 ▸), mol­ecules form a columnar head-to-tail packing parallel to [010] *via* van der Waals inter­actions, with the columns held together by weak C—H⋯π contacts between the H16*B* atom and the centroid of the C1–C6 ring [2.862 (19) Å, 125.4 (10)°] and between the H17*B* atom and the centroid of the C8–C13 ring [2.97 (2) Å, 120.1 (12)°].

**Figure 6:**
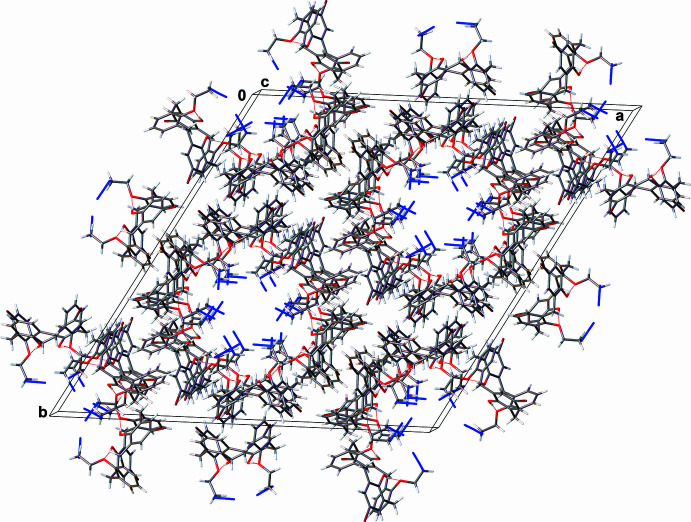
Fragment of the crystal packing of 25,27-bis­(2-azido­eth­oxy)-5,17-di­bromo-26,28-di­hydroxy­calix[4]arene (**3**).

In the crystal structure of **3** (Fig. 6 ▸), C—H⋯π contacts between the H31*A* atom and the centroid of the C1–C5/C25 ring [2.6130 (11) Å, 122.1 (2)°] and between the H29*B* atom and the centroid of the C13–C17/C27 ring [2.8400 (11) Å, 129.56 (18)°] organize mol­ecules into large channels passing parallel to the *c* axis, which are filled by highly disordered CH_2_Cl_2_ solvent mol­ecules. According to the applied SQUEEZE procedure (Spek, 2015 ▸), the solvent-accessible void volume is as large as 585 Å^3^ per unit cell and contains fragments with an electron count of 171 e^−^. This correspond to about 4.5 CH_2_Cl_2_ mol­ecules in the unit cell, or 0.25 CH_2_Cl_2_ mol­ecules per formula unit. The nitro­gen atoms of the azide groups have comparatively large displacement parameters because these groups are directed into the channels and do not participate in any strong inter­molecular inter­actions. Adjacent channels are assembled into the tri-periodic structure by π–π [*d*(C21⋯C19-C23/C28_centroid_) = 3.446 (4) Å, centroid-to-centroid shift of 2.533 (4) Å] and C—H⋯π inter­actions between the H20 atom and the centroid of the C1–C5/C25 ring [3.0326 (11) Å, 154.5 (2)°] and between the H22 atom and the centroid of the C13–C17/C27 ring [3.6003 (11) Å, 152.69 (18)°].

## 4. Database survey

The crystal structures of more than 750 calix[4]arenes have been published so far, as revealed by a search of the Cambridge Structural Database (CSD, version 5.45, updated to November 2023; Groom *et al.*, 2016 ▸). The database analysis shows that calix[4]arenes, which are distally disubstituted at the lower rim, prefer a pinched cone conformation in solution and in the solid state, which agrees well with the result of the present study. In addition, there are three thia­calix[4]arenes having OH groups in distal positions of the lower rim: JIPQIJ01 (Dvořáková *et al.*, 2007 ▸); KURKAL, KURKEP (Wang *et al.*, 2015 ▸), which are isostructural with compound **3**. Several crystal structures of di­bromo-substituted calix[4]arene 1,3-alternates have also been reported, in which the Br⋯Br distance varies from 3.967 (5) Å (BAGYAJ; Krebs *et al.*, 1998 ▸) to 4.112 (8) Å (KARNAT; Sykora *et al.*, 2005 ▸).

## 5. Synthesis and crystallization

The title compounds were prepared as follows:

**5,17-Di­bromo-26,28-dihy­droxy-25,27-dipropynyloxycalix[4]arene (1) (** **cone** **)**

To a stirred solution of 26,28-dihy­droxy-25,27-dipropynyloxycalix[4]arene (0.50 mg, 1.0 mmol) (Xu *et al.*, 1996 ▸) in di­chloro­methane *N*-bromo­succinimide (0.39 g, 2.2 mmol) was added and the resultant mixture stirred at 298 K for 24 h. The solvent was removed under reduced pressure and the residue was purified by flash chromatography (silica, di­chloro­methane) followed by crystallization from a di­chloro­methane/methanol solvent mixture. Single crystals suitable for X-ray analysis were grown by slow evaporation of the solvent from a solution of the substance in a CH_2_Cl_2_/MeOH mixture (1:1 *v*/*v*). Yield 0.55 g (77%). M.p. 515–516 K. ESI-MS: *m*/*z*: 676.0516 [*M* + NH_4_]^+^ for C_34_H_30_Br_2_NO_4_ (676.0516). ^1^H NMR (CDCl_3_, 400 MHz): δ = 7.19 (*s*, 4H; ArH), 7.15 (*s*, 2H; OH), 6.88–6.83 (*m*, 4H; ArH), 6.80–6.75 (*m*, 2H; ArH), 4.76 (*d*, 4H, ^4^ *J*_HH_ = 2.4 Hz; OCH_2_), 4.34 (*d*, 4H, ^2^ *J*_HH_ = 13.4 Hz; ArCH_2_Ar), 3.35 (*d*, 4H, ^2^ *J*_HH_ =13.4 Hz; ArCH_2_Ar), 2.58 (*t*, 2H, ^4^ *J*_HH_ = 2.4 Hz; CH) ppm; ^13^C NMR (100 MHz, CDCl_3_): δ = 152.12, 151.26, 132.62 (C_Ar_), 130.83 (CH_Ar_), 130.03 (C_Ar_), 129.29, 125.91 (CH_Ar_), 110.76 (C_Ar_), 77.92 (CCH), 76.95 (CCH), 63.50 (OCH_2_), 31.51 (ArCH_2_Ar) ppm.

**5,17-Di­bromo-26,28-dipropoxy-25,27-dipropynyloxycalix[4]arene (2) (** **1,3-alternate** **)**

A mixture of calix[4]arene **1** (0.45g, 0.7 mmol) and anhydrous Cs_2_CO_3_ (0.90 g, 1.8 mmol) in dry DMF (15 ml) was stirred at room temperature for 2 h. 1-Iodo­propane (0.40 ml, 4.1 mmol) was added and the mixture stirred for 48 h at 298 K. The solvent was removed under reduced pressure with heating below 333 K, and the residue was parted between di­chloro­methane and 2M HCl. The organic layer was separated, washed with water, dried with MgSO_4_ and concentrated to dryness. The residue was purified by flash chromatography (silica, gradient from hexane to hexa­ne/di­chloro­methane (1:1)). Single crystals suitable for X-ray analysis were grown by slow evaporation of the solvent from a solution of the substance in a CH_2_Cl_2_/MeOH mixture (1:1 *v*/*v*). Yield 0.16 g (31%). M.p. 478–479 K. ESI-MS: *m*/*z*: 760.1462 [*M* + NH_4_]^+^ for C_40_H_42_Br_2_NO_4_ (760.1455). ^1^H NMR (CDCl_3_, 400 MHz): δ = 7.28 (*s*, 4H; ArH), 6.97 (*d*, 4H, ^3^ *J*_HH_ = 7.6 Hz; ArH), 6.72 (*t*, 2H, ^3^ *J*_HH_ = 7.6Hz; ArH), 4.22 (*d*, 4H, ^4^ *J*_HH_ = 2.4 Hz; OCH_2_CCH), 3.66 (*d*, 4H, ^2^ *J*_HH_ = 14.5 Hz; ArCH_2_Ar), 3.57 (*d*, 4H, ^2^ *J*_HH_ = 14.5 Hz; ArCH_2_Ar), 3.49–3.43 (*m*, 4H; OCH_2_CH_2_), 2.55 (*t*, 2H, ^4^ *J*_HH_ = 2.4 Hz; CCH), 1.67–1.57 (*m*, 4H; CH_2_CH_3_), 0.88 (*t*, 6H, ^3^ *J*_HH_ = 7.5 Hz; CH_3_) ppm; ^13^C NMR (100 MHz, CDCl_3_): δ = 155.40, 155.07, 135.09, 133.59 (C_Ar_), 132.88, 130.15, 122.58 (CH_Ar_), 114.72 (C_Ar_), 79.48 (CCH), 75.14 (CCH), 73.68 (OCH_2_CH_2_), 58.77 (OCH_2_CCH), 36.25 (ArCH_2_Ar), 23.53 (CH_2_CH_3_), 10.34 (CH_3_) ppm.

**25,27-Bis­(2-azido­eth­oxy)-5,17-di­bromo-26,28-di­hydroxy­calix[4]arene (3) (** **cone** **)**

To a stirred solution of 25,27-bis­(2-azido­eth­oxy)-26,28-di­hydroxy­calix[4]arene (0.56 mg, 1.0 mmol) (Gorbunov *et al.*, 2021 ▸) in di­chloro­methane *N*-bromo­succinimide (0.39 g, 2.2 mmol) was added and the resultant mixture stirred at 298 K for 24 h. The solvent was removed under reduced pressure and the residue was purified by flash chromatography (silica, di­chloro­methane) followed by crystallization from di­chloro­methane/methanol solvent mixture. Single crystals suitable for X-ray analysis were grown by slow evaporation of the solvent from a solution of the substance in a CH_2_Cl_2_/MeOH mixture (1:1 *v*/*v*). Yield 0.63g (88%). M.p. 538–539 K. ESI-MS: *m*/*z*: 738.0861 [*M* + NH_4_]^+^ for C_32_H_32_Br_2_N_7_O_4_ (738.0857). ^1^H NMR (CDCl_3_, 400 MHz): δ = 7.57 (*s*, 2H; OH), 7.19 (*s*, 4H; ArH), 6.91–6.86 (*m*, 4H; ArH), 6.81–6.76 (*m*, 2H; ArH), 4.29 (*d*, 4H, ^2^ *J*_HH_ = 13.2 Hz; ArCH_2_Ar), 4.07–4.02 (*m*, 4H; CH_2_CH_2_), 3.88–3.83 (*m*, 4H; CH_2_CH_2_), 3.34 (*d*, 4H, ^2^ *J*_HH_ = 13.2 Hz; ArCH_2_Ar) ppm; ^13^C NMR (100 MHz, CDCl_3_): δ = 152.40, 151.43, 132.23 (C_Ar_), 130.84 (CH_Ar_), 129.79 (C_Ar_), 129.40, 125.77 (CH_Ar_), 110.53 (C_Ar_), 74.41 (OCH_2_), 51.07 (CH_2_N_3_), 30.90 (ArCH_2_Ar) ppm.

## 6. Refinement

Crystal data, data collection and structure refinement details are summarized in Table 3 ▸. All C-bound hydrogen atoms in the structures of **1** and **3** were placed in calculated positions and refined using a riding model [C—H = 0.94–0.97 Å with *U*_iso_(H) = 1.2–1.5*U*_eq_(C)]. Hydrogen atoms of hy­droxy groups were located from difference electron-density maps and were refined with *U*_iso_(H) = 1.5*U*_eq_(O). In the structure of **2**, hydrogen atoms were located from difference electron-density maps and were refined freely. In the structure of **1**, one bromine atom was found to be disordered over two positions with a refined occupancy ratio of 0.928 (5):0.072 (5). In the structure of **3**, highly disordered solvent CH_2_Cl_2_ mol­ecules are present. Their contributions to the intensity data was removed by the SQUEEZE procedure (Spek, 2015 ▸) as implemented in the *OLEX2* package (Dolomanov *et al.*, 2009 ▸). The SIMU instruction was used to restrain the *U* ^ij^ components of the disordered bromine atoms in the structure of **1** and nitro­gen atoms in the structure of **3**. The most disagreeable reflections with an error/s.u. of more than 10 (100 in the data for **1**; 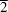 40, 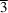 60 and 030 in the data for **3**) were omitted using the OMIT instruction in *SHELXL* (Sheldrick, 2015*b* ▸).

## Supplementary Material

Crystal structure: contains datablock(s) 1, 2, 3. DOI: 10.1107/S2056989024003785/wm5716sup1.cif

Structure factors: contains datablock(s) 1. DOI: 10.1107/S2056989024003785/wm57161sup2.hkl

Supporting information file. DOI: 10.1107/S2056989024003785/wm57161sup5.mol

Structure factors: contains datablock(s) 2. DOI: 10.1107/S2056989024003785/wm57162sup3.hkl

Supporting information file. DOI: 10.1107/S2056989024003785/wm57162sup6.mol

Structure factors: contains datablock(s) 3. DOI: 10.1107/S2056989024003785/wm57163sup4.hkl

Supporting information file. DOI: 10.1107/S2056989024003785/wm57163sup7.mol

Synthesis and NMR spectra. DOI: 10.1107/S2056989024003785/wm5716sup8.pdf

CCDC references: 2350927, 2350926, 2350925

Additional supporting information:  crystallographic information; 3D view; checkCIF report

## Figures and Tables

**Figure 2:**
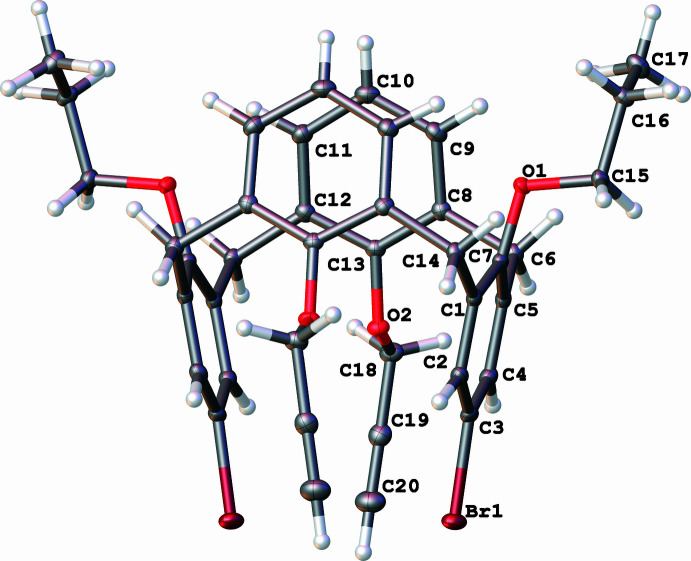
Mol­ecular structure of 5,17-di­bromo-26,28-dipropoxy-25,27-dipropynyloxycalix[4]arene (**2**), with displacement ellipsoids drawn at the 50% probability level.

**Table 1:** Hydrogen-bond geometry (Å, °) for **1** [Chem scheme1]

*D*—H⋯*A*	*D*—H	H⋯*A*	*D*⋯*A*	*D*—H⋯*A*
O1—H1⋯O4	0.84	1.81	2.6270 (18)	165
O3—H3⋯O2	0.84	2.02	2.8207 (18)	160

**Table 2:** Hydrogen-bond geometry (Å, °) for **3** [Chem scheme1]

*D*—H⋯*A*	*D*—H	H⋯*A*	*D*⋯*A*	*D*—H⋯*A*
O3—H3⋯O4	0.84	1.87	2.691 (3)	164
O1—H1⋯O2	0.84	1.95	2.764 (3)	162

**Table 3:** Experimental details

	**1**	**2**	**3**
Crystal data			
Chemical formula	C_34_H_26_Br_2_O_4_	C_40_H_38_Br_2_O_4_	C_32_H_28_Br_2_N_6_O_4_
*M*_r_	658.37	742.52	720.42
Crystal system, space group	Triclinic, *P* 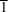	Orthorhombic, *P* *b* *c* *n*	Trigonal, *R* 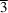
Temperature (K)	100	100	100
*a*, *b*, *c* (Å)	10.1542 (3), 11.9156 (3), 11.9964 (4)	18.1223 (7), 9.9840 (4), 18.2863 (7)	36.3261 (7), 36.3261 (7), 12.1054 (4)
α, β, γ (°)	75.221 (1), 88.341 (1), 81.751 (1)	90, 90, 90	90, 90, 120
*V* (Å^3^)	1388.90 (7)	3308.6 (2)	13834.0 (7)
*Z*	2	4	18
Radiation type	Mo *K*α	Mo *K*α	Mo *K*α
μ (mm^−1^)	2.96	2.49	2.69
Crystal size (mm)	0.32 × 0.16 × 0.12	0.35 × 0.25 × 0.22	0.37 × 0.13 × 0.1

Data collection			
Diffractometer	Bruker D8 Venture	Bruker D8 Venture	Bruker D8 Venture
Absorption correction	Multi-scan (*SADABS*; Krause *et al.,* 2015 ▸)	Multi-scan (*SADABS*; Krause *et al.*, 2015 ▸)	Multi-scan (*SADABS*; Krause *et al.*, 2015 ▸)
*T*_min_, *T*_max_	0.501, 0.746	0.545, 0.746	0.516, 0.746
No. of measured, independent and observed [*I* > 2σ(*I*)] reflections	31619, 8481, 6661	55960, 5529, 4481	45251, 7804, 6043
*R*_int_	0.043	0.060	0.047
(sin θ/λ)_max_ (Å^−1^)	0.715	0.736	0.671

Refinement			
*R*[*F* ^2^ > 2σ(*F* ^2^)], *wR*(*F* ^2^), *S*	0.036, 0.087, 1.04	0.030, 0.069, 1.04	0.040, 0.097, 1.03
No. of reflections	8481	5529	7804
No. of parameters	373	283	399
No. of restraints	7	0	24
H-atom treatment	H-atom parameters constrained	Only H-atom coordinates refined	H-atom parameters constrained
Δρ_max_, Δρ_min_ (e Å^−3^)	0.49, −0.80	0.42, −0.51	1.30, −1.12

Computer programs: *APEX3* and *SAINT* (Bruker, 2017 ▸), *SHELXT* (Sheldrick, 2015*a* ▸), *SHELXL* (Sheldrick, 2015*b* ▸) and *OLEX2* (Dolomanov *et al.*, 2009 ▸).

## supplementary crystallographic information

### 5,17-Dibromo-26,28-dihydroxy-25,27-dipropynyloxycalix[4]arene (1) Crystal data

**Table:**

C_34_H_26_Br_2_O_4_	*Z* = 2
*M**_r_* = 658.37	*F*(000) = 664
Triclinic, *P*1	*D*_x_ = 1.574 Mg m^−^^3^
*a* = 10.1542 (3) Å	Mo *K*α radiation, λ = 0.71073 Å
*b* = 11.9156 (3) Å	Cell parameters from 9976 reflections
*c* = 11.9964 (4) Å	θ = 2.5–30.5°
α = 75.221 (1)°	µ = 2.96 mm^−^^1^
β = 88.341 (1)°	*T* = 100 K
γ = 81.751 (1)°	Block, yellow
*V* = 1388.90 (7) Å^3^	0.32 × 0.16 × 0.12 mm

### 5,17-Dibromo-26,28-dihydroxy-25,27-dipropynyloxycalix[4]arene (1) Data collection

**Table:**

Bruker D8 Venture diffractometer	8481 independent reflections
Radiation source: microfocus sealed X-ray tube, Incoatec IµS microsource	6661 reflections with *I* > 2σ(*I*)
Focusing mirrors monochromator	*R*_int_ = 0.043
Detector resolution: 10.4 pixels mm^-1^	θ_max_ = 30.6°, θ_min_ = 1.8°
ω–scan	*h* = −14→14
Absorption correction: multi-scan (*SADABS*; Krause *et al.,* 2015)	*k* = −17→17
*T*_min_ = 0.501, *T*_max_ = 0.746	*l* = −16→17
31619 measured reflections	

### 5,17-Dibromo-26,28-dihydroxy-25,27-dipropynyloxycalix[4]arene (1) Refinement

**Table:**

Refinement on *F*^2^	7 restraints
Least-squares matrix: full	Hydrogen site location: inferred from neighbouring sites
*R*[*F*^2^ > 2σ(*F*^2^)] = 0.036	H-atom parameters constrained
*wR*(*F*^2^) = 0.087	*w* = 1/[σ^2^(*F*_o_^2^) + (0.0365*P*)^2^ + 0.736*P*] where *P* = (*F*_o_^2^ + 2*F*_c_^2^)/3
*S* = 1.04	(Δ/σ)_max_ = 0.001
8481 reflections	Δρ_max_ = 0.49 e Å^−^^3^
373 parameters	Δρ_min_ = −0.80 e Å^−^^3^

### 5,17-Dibromo-26,28-dihydroxy-25,27-dipropynyloxycalix[4]arene (1) Special details

**Table:**

Geometry. All esds (except the esd in the dihedral angle between two l.s. planes) are estimated using the full covariance matrix. The cell esds are taken into account individually in the estimation of esds in distances, angles and torsion angles; correlations between esds in cell parameters are only used when they are defined by crystal symmetry. An approximate (isotropic) treatment of cell esds is used for estimating esds involving l.s. planes.

### 5,17-Dibromo-26,28-dihydroxy-25,27-dipropynyloxycalix[4]arene (1) Fractional atomic coordinates and isotropic or equivalent isotropic displacement parameters (Å^2^)

**Table:**

	*x*	*y*	*z*	*U*_iso_*/*U*_eq_	Occ. (<1)
Br1	0.13667 (2)	0.10801 (2)	0.34716 (2)	0.02325 (6)	
Br3	0.4344 (16)	0.8992 (4)	0.7623 (12)	0.0478 (18)	0.072 (5)
O1	0.37255 (13)	0.20076 (13)	0.76345 (11)	0.0175 (3)	
H1	0.455133	0.201641	0.759281	0.026*	
O4	0.62310 (13)	0.22902 (12)	0.77582 (11)	0.0147 (3)	
O2	0.21281 (13)	0.28716 (12)	0.93959 (11)	0.0149 (3)	
O3	0.45446 (13)	0.37816 (12)	0.88209 (12)	0.0175 (3)	
H3	0.381434	0.360749	0.910983	0.026*	
C1	0.40950 (19)	0.16828 (16)	0.57182 (16)	0.0142 (4)	
C25	0.32705 (19)	0.18609 (16)	0.66343 (16)	0.0140 (4)	
C2	0.3504 (2)	0.14541 (16)	0.47770 (16)	0.0164 (4)	
H2	0.404008	0.131552	0.415021	0.020*	
C23	0.60857 (18)	0.28084 (16)	0.57005 (16)	0.0142 (4)	
C26	0.14200 (18)	0.37135 (17)	0.85034 (16)	0.0144 (4)	
C5	0.18917 (19)	0.18555 (17)	0.65907 (16)	0.0158 (4)	
C28	0.63632 (18)	0.31164 (16)	0.67091 (16)	0.0136 (4)	
C4	0.1333 (2)	0.16353 (17)	0.56418 (17)	0.0177 (4)	
H4	0.040104	0.162716	0.560404	0.021*	
C20	0.68421 (18)	0.49935 (17)	0.56564 (16)	0.0155 (4)	
H20	0.707334	0.574420	0.563143	0.019*	
C7	0.08651 (18)	0.33525 (18)	0.76248 (16)	0.0159 (4)	
C27	0.45330 (19)	0.49577 (16)	0.85977 (16)	0.0144 (4)	
C3	0.2149 (2)	0.14269 (17)	0.47470 (16)	0.0173 (4)	
C33	0.70622 (19)	0.05765 (18)	0.91211 (17)	0.0183 (4)	
C30	0.0378 (2)	0.18942 (18)	1.04971 (17)	0.0183 (4)	
C19	0.66961 (18)	0.42045 (17)	0.67255 (16)	0.0137 (4)	
C16	0.5776 (2)	0.65712 (18)	0.79072 (16)	0.0182 (4)	
H16	0.656387	0.687064	0.759724	0.022*	
C13	0.34441 (19)	0.57215 (17)	0.88396 (16)	0.0153 (4)	
C11	0.13953 (18)	0.48938 (17)	0.84800 (16)	0.0151 (4)	
C32	0.74233 (19)	0.14878 (18)	0.81472 (17)	0.0174 (4)	
H32A	0.810036	0.190259	0.838597	0.021*	
H32B	0.779649	0.113938	0.751985	0.021*	
C22	0.62569 (19)	0.36187 (17)	0.46568 (16)	0.0163 (4)	
H22	0.610029	0.343350	0.395175	0.020*	
C6	0.10297 (19)	0.20789 (18)	0.75864 (17)	0.0182 (4)	
H6A	0.014234	0.185145	0.751264	0.022*	
H6B	0.143676	0.158222	0.831971	0.022*	
C12	0.21709 (19)	0.52806 (18)	0.93450 (17)	0.0163 (4)	
H12A	0.160564	0.591182	0.960687	0.020*	
H12B	0.239323	0.461186	1.002593	0.020*	
C8	0.02054 (19)	0.42259 (19)	0.67312 (17)	0.0196 (4)	
H8	−0.019001	0.400917	0.612324	0.023*	
C24	0.56005 (19)	0.16485 (17)	0.57366 (17)	0.0161 (4)	
H24A	0.596010	0.106554	0.644376	0.019*	
H24B	0.597657	0.136945	0.506871	0.019*	
C29	0.1451 (2)	0.26107 (18)	1.04853 (16)	0.0185 (4)	
H29A	0.106800	0.335589	1.066540	0.022*	
H29B	0.210651	0.218888	1.109793	0.022*	
C34	0.6739 (2)	−0.01519 (19)	0.99099 (19)	0.0218 (4)	
H34	0.647887	−0.073636	1.054290	0.026*	
C21	0.66540 (19)	0.46942 (17)	0.46356 (17)	0.0168 (4)	
H21	0.679652	0.522512	0.391855	0.020*	
C14	0.3541 (2)	0.69105 (18)	0.86054 (17)	0.0186 (4)	
H14	0.280958	0.744225	0.875755	0.022*	
C15	0.4698 (2)	0.73211 (17)	0.81518 (17)	0.0202 (4)	
C17	0.57032 (19)	0.53733 (17)	0.81171 (16)	0.0143 (4)	
C18	0.68642 (18)	0.45380 (18)	0.78479 (16)	0.0159 (4)	
H18A	0.697858	0.381654	0.848659	0.019*	
H18B	0.768496	0.490555	0.780692	0.019*	
C9	0.0116 (2)	0.5402 (2)	0.67141 (18)	0.0219 (4)	
H9	−0.036051	0.598057	0.611156	0.026*	
C31	−0.0464 (2)	0.13008 (19)	1.05713 (18)	0.0224 (4)	
H31	−0.114571	0.082004	1.063136	0.027*	
C10	0.07224 (19)	0.57330 (18)	0.75746 (17)	0.0188 (4)	
H10	0.067891	0.654129	0.754765	0.023*	
Br2	0.48141 (12)	0.89367 (4)	0.79296 (8)	0.03315 (19)	0.928 (5)

### 5,17-Dibromo-26,28-dihydroxy-25,27-dipropynyloxycalix[4]arene (1) Atomic displacement parameters (Å^2^)

**Table:**

	*U*^11^	*U*^22^	*U*^33^	*U*^12^	*U*^13^	*U*^23^
Br1	0.02602 (12)	0.02894 (12)	0.01807 (10)	−0.00387 (9)	−0.00172 (8)	−0.01170 (8)
Br3	0.069 (4)	0.023 (2)	0.058 (3)	−0.023 (2)	0.021 (3)	−0.015 (2)
O1	0.0128 (6)	0.0278 (8)	0.0150 (6)	−0.0074 (6)	0.0039 (5)	−0.0092 (6)
O4	0.0113 (6)	0.0160 (7)	0.0140 (6)	0.0000 (5)	0.0021 (5)	0.0003 (5)
O2	0.0143 (6)	0.0176 (7)	0.0125 (6)	−0.0027 (5)	0.0027 (5)	−0.0032 (5)
O3	0.0125 (6)	0.0134 (6)	0.0264 (7)	−0.0036 (5)	0.0043 (5)	−0.0044 (6)
C1	0.0163 (9)	0.0103 (8)	0.0159 (8)	−0.0030 (7)	0.0038 (7)	−0.0028 (7)
C25	0.0166 (9)	0.0129 (8)	0.0135 (8)	−0.0043 (7)	0.0020 (7)	−0.0044 (7)
C2	0.0223 (10)	0.0132 (9)	0.0138 (8)	−0.0022 (7)	0.0042 (7)	−0.0041 (7)
C23	0.0100 (8)	0.0152 (9)	0.0176 (9)	−0.0016 (7)	0.0032 (7)	−0.0049 (7)
C26	0.0095 (8)	0.0197 (9)	0.0139 (8)	−0.0013 (7)	0.0025 (7)	−0.0044 (7)
C5	0.0157 (9)	0.0169 (9)	0.0164 (9)	−0.0050 (7)	0.0034 (7)	−0.0061 (7)
C28	0.0105 (8)	0.0150 (9)	0.0139 (8)	−0.0013 (7)	0.0028 (6)	−0.0015 (7)
C4	0.0167 (9)	0.0193 (10)	0.0186 (9)	−0.0044 (8)	0.0008 (7)	−0.0065 (8)
C20	0.0111 (8)	0.0163 (9)	0.0189 (9)	−0.0036 (7)	0.0017 (7)	−0.0032 (7)
C7	0.0099 (8)	0.0238 (10)	0.0161 (9)	−0.0045 (7)	0.0052 (7)	−0.0083 (8)
C27	0.0160 (9)	0.0144 (9)	0.0130 (8)	−0.0040 (7)	−0.0017 (7)	−0.0026 (7)
C3	0.0238 (10)	0.0158 (9)	0.0144 (9)	−0.0040 (8)	−0.0009 (7)	−0.0066 (7)
C33	0.0144 (9)	0.0186 (10)	0.0210 (9)	0.0015 (7)	−0.0021 (7)	−0.0052 (8)
C30	0.0202 (10)	0.0202 (10)	0.0144 (9)	−0.0010 (8)	0.0035 (7)	−0.0057 (8)
C19	0.0085 (8)	0.0178 (9)	0.0146 (8)	−0.0031 (7)	0.0014 (6)	−0.0033 (7)
C16	0.0197 (10)	0.0218 (10)	0.0159 (9)	−0.0110 (8)	0.0019 (7)	−0.0056 (8)
C13	0.0170 (9)	0.0166 (9)	0.0137 (8)	−0.0038 (7)	0.0003 (7)	−0.0057 (7)
C11	0.0099 (8)	0.0201 (9)	0.0160 (9)	−0.0017 (7)	0.0035 (7)	−0.0065 (7)
C32	0.0122 (9)	0.0196 (10)	0.0177 (9)	0.0010 (7)	0.0012 (7)	−0.0017 (8)
C22	0.0152 (9)	0.0190 (9)	0.0151 (9)	−0.0033 (7)	0.0022 (7)	−0.0050 (7)
C6	0.0139 (9)	0.0255 (10)	0.0196 (9)	−0.0082 (8)	0.0046 (7)	−0.0113 (8)
C12	0.0146 (9)	0.0183 (9)	0.0177 (9)	−0.0018 (7)	0.0024 (7)	−0.0081 (8)
C8	0.0096 (9)	0.0335 (12)	0.0170 (9)	−0.0007 (8)	0.0011 (7)	−0.0104 (8)
C24	0.0156 (9)	0.0150 (9)	0.0188 (9)	−0.0023 (7)	0.0048 (7)	−0.0065 (7)
C29	0.0200 (10)	0.0231 (10)	0.0145 (9)	−0.0068 (8)	0.0044 (7)	−0.0070 (8)
C34	0.0190 (10)	0.0203 (10)	0.0237 (10)	−0.0008 (8)	0.0005 (8)	−0.0025 (8)
C21	0.0137 (9)	0.0185 (9)	0.0163 (9)	−0.0032 (7)	0.0028 (7)	−0.0006 (7)
C14	0.0225 (10)	0.0169 (9)	0.0183 (9)	−0.0027 (8)	0.0036 (8)	−0.0080 (8)
C15	0.0315 (11)	0.0154 (9)	0.0168 (9)	−0.0090 (8)	0.0040 (8)	−0.0068 (8)
C17	0.0145 (9)	0.0178 (9)	0.0118 (8)	−0.0042 (7)	0.0000 (7)	−0.0046 (7)
C18	0.0112 (9)	0.0222 (10)	0.0154 (9)	−0.0040 (7)	0.0004 (7)	−0.0058 (8)
C9	0.0131 (9)	0.0290 (11)	0.0195 (10)	0.0040 (8)	−0.0006 (7)	−0.0025 (9)
C31	0.0180 (10)	0.0266 (11)	0.0243 (10)	−0.0057 (8)	0.0042 (8)	−0.0087 (9)
C10	0.0155 (9)	0.0194 (10)	0.0203 (9)	0.0015 (8)	0.0048 (7)	−0.0053 (8)
Br2	0.0503 (4)	0.01800 (14)	0.0381 (3)	−0.01664 (17)	0.0244 (2)	−0.01573 (14)

### 5,17-Dibromo-26,28-dihydroxy-25,27-dipropynyloxycalix[4]arene (1) Geometric parameters (Å, º)

**Table:**

Br1—C3	1.9052 (19)	C30—C31	1.173 (3)
Br3—C15	1.913 (5)	C19—C18	1.519 (3)
O1—H1	0.8400	C16—H16	0.9500
O1—C25	1.359 (2)	C16—C15	1.385 (3)
O4—C28	1.403 (2)	C16—C17	1.397 (3)
O4—C32	1.439 (2)	C13—C12	1.517 (3)
O2—C26	1.400 (2)	C13—C14	1.390 (3)
O2—C29	1.442 (2)	C11—C12	1.518 (3)
O3—H3	0.8400	C11—C10	1.393 (3)
O3—C27	1.356 (2)	C32—H32A	0.9900
C1—C25	1.403 (3)	C32—H32B	0.9900
C1—C2	1.397 (3)	C22—H22	0.9500
C1—C24	1.524 (3)	C22—C21	1.392 (3)
C25—C5	1.403 (3)	C6—H6A	0.9900
C2—H2	0.9500	C6—H6B	0.9900
C2—C3	1.383 (3)	C12—H12A	0.9900
C23—C28	1.398 (3)	C12—H12B	0.9900
C23—C22	1.396 (3)	C8—H8	0.9500
C23—C24	1.523 (3)	C8—C9	1.386 (3)
C26—C7	1.396 (3)	C24—H24A	0.9900
C26—C11	1.396 (3)	C24—H24B	0.9900
C5—C4	1.385 (3)	C29—H29A	0.9900
C5—C6	1.516 (3)	C29—H29B	0.9900
C28—C19	1.391 (3)	C34—H34	0.9500
C4—H4	0.9500	C21—H21	0.9500
C4—C3	1.388 (3)	C14—H14	0.9500
C20—H20	0.9500	C14—C15	1.381 (3)
C20—C19	1.402 (3)	C15—Br2	1.895 (2)
C20—C21	1.385 (3)	C17—C18	1.513 (3)
C7—C6	1.515 (3)	C18—H18A	0.9900
C7—C8	1.398 (3)	C18—H18B	0.9900
C27—C13	1.401 (3)	C9—H9	0.9500
C27—C17	1.405 (3)	C9—C10	1.384 (3)
C33—C32	1.456 (3)	C31—H31	0.9500
C33—C34	1.184 (3)	C10—H10	0.9500
C30—C29	1.475 (3)		
			
C25—O1—H1	109.5	H32A—C32—H32B	108.5
C28—O4—C32	114.33 (14)	C23—C22—H22	119.6
C26—O2—C29	115.53 (14)	C21—C22—C23	120.87 (18)
C27—O3—H3	109.5	C21—C22—H22	119.6
C25—C1—C24	123.34 (17)	C5—C6—H6A	109.2
C2—C1—C25	117.70 (17)	C5—C6—H6B	109.2
C2—C1—C24	118.84 (17)	C7—C6—C5	112.17 (16)
O1—C25—C1	123.84 (17)	C7—C6—H6A	109.2
O1—C25—C5	114.80 (16)	C7—C6—H6B	109.2
C1—C25—C5	121.32 (17)	H6A—C6—H6B	107.9
C1—C2—H2	119.6	C13—C12—C11	112.16 (15)
C3—C2—C1	120.79 (18)	C13—C12—H12A	109.2
C3—C2—H2	119.6	C13—C12—H12B	109.2
C28—C23—C24	121.58 (17)	C11—C12—H12A	109.2
C22—C23—C28	117.03 (17)	C11—C12—H12B	109.2
C22—C23—C24	121.38 (17)	H12A—C12—H12B	107.9
C7—C26—O2	119.17 (17)	C7—C8—H8	119.3
C7—C26—C11	122.50 (17)	C9—C8—C7	121.32 (19)
C11—C26—O2	118.10 (16)	C9—C8—H8	119.3
C25—C5—C6	119.75 (17)	C1—C24—H24A	108.4
C4—C5—C25	119.64 (17)	C1—C24—H24B	108.4
C4—C5—C6	120.61 (17)	C23—C24—C1	115.60 (16)
C23—C28—O4	117.21 (17)	C23—C24—H24A	108.4
C19—C28—O4	119.01 (16)	C23—C24—H24B	108.4
C19—C28—C23	123.71 (17)	H24A—C24—H24B	107.4
C5—C4—H4	120.4	O2—C29—C30	113.22 (16)
C5—C4—C3	119.26 (18)	O2—C29—H29A	108.9
C3—C4—H4	120.4	O2—C29—H29B	108.9
C19—C20—H20	119.5	C30—C29—H29A	108.9
C21—C20—H20	119.5	C30—C29—H29B	108.9
C21—C20—C19	120.95 (18)	H29A—C29—H29B	107.7
C26—C7—C6	122.24 (17)	C33—C34—H34	180.0
C26—C7—C8	117.23 (19)	C20—C21—C22	120.23 (18)
C8—C7—C6	120.43 (18)	C20—C21—H21	119.9
O3—C27—C13	123.07 (17)	C22—C21—H21	119.9
O3—C27—C17	115.61 (17)	C13—C14—H14	119.9
C13—C27—C17	121.31 (18)	C15—C14—C13	120.17 (19)
C2—C3—Br1	120.20 (15)	C15—C14—H14	119.9
C2—C3—C4	121.25 (18)	C16—C15—Br3	128.5 (3)
C4—C3—Br1	118.55 (15)	C16—C15—Br2	120.16 (15)
C34—C33—C32	178.5 (2)	C14—C15—Br3	108.5 (4)
C31—C30—C29	176.3 (2)	C14—C15—C16	121.34 (18)
C28—C19—C20	117.00 (17)	C14—C15—Br2	118.44 (16)
C28—C19—C18	121.74 (16)	C27—C17—C18	120.63 (17)
C20—C19—C18	121.25 (17)	C16—C17—C27	118.52 (18)
C15—C16—H16	120.1	C16—C17—C18	120.85 (17)
C15—C16—C17	119.89 (18)	C19—C18—H18A	108.9
C17—C16—H16	120.1	C19—C18—H18B	108.9
C27—C13—C12	121.59 (17)	C17—C18—C19	113.51 (16)
C14—C13—C27	118.76 (18)	C17—C18—H18A	108.9
C14—C13—C12	119.65 (17)	C17—C18—H18B	108.9
C26—C11—C12	122.03 (17)	H18A—C18—H18B	107.7
C10—C11—C26	118.15 (18)	C8—C9—H9	120.0
C10—C11—C12	119.61 (18)	C10—C9—C8	119.99 (19)
O4—C32—C33	107.29 (15)	C10—C9—H9	120.0
O4—C32—H32A	110.3	C30—C31—H31	180.0
O4—C32—H32B	110.3	C11—C10—H10	119.7
C33—C32—H32A	110.3	C9—C10—C11	120.69 (19)
C33—C32—H32B	110.3	C9—C10—H10	119.7
			
O1—C25—C5—C4	−175.79 (17)	C27—C13—C12—C11	−70.9 (2)
O1—C25—C5—C6	3.6 (3)	C27—C13—C14—C15	−0.6 (3)
O4—C28—C19—C20	178.82 (16)	C27—C17—C18—C19	80.0 (2)
O4—C28—C19—C18	−2.5 (3)	C19—C20—C21—C22	2.9 (3)
O2—C26—C7—C6	−1.5 (3)	C16—C17—C18—C19	−100.8 (2)
O2—C26—C7—C8	−177.89 (16)	C13—C27—C17—C16	1.5 (3)
O2—C26—C11—C12	3.5 (3)	C13—C27—C17—C18	−179.27 (17)
O2—C26—C11—C10	178.24 (16)	C13—C14—C15—Br3	167.1 (6)
O3—C27—C13—C12	−1.7 (3)	C13—C14—C15—C16	0.8 (3)
O3—C27—C13—C14	178.42 (17)	C13—C14—C15—Br2	−176.50 (15)
O3—C27—C17—C16	−177.58 (16)	C11—C26—C7—C6	172.93 (17)
O3—C27—C17—C18	1.6 (3)	C11—C26—C7—C8	−3.5 (3)
C1—C25—C5—C4	2.0 (3)	C32—O4—C28—C23	89.9 (2)
C1—C25—C5—C6	−178.60 (18)	C32—O4—C28—C19	−93.1 (2)
C1—C2—C3—Br1	−178.64 (14)	C22—C23—C28—O4	−178.14 (16)
C1—C2—C3—C4	0.6 (3)	C22—C23—C28—C19	5.0 (3)
C25—C1—C2—C3	1.2 (3)	C22—C23—C24—C1	−86.3 (2)
C25—C1—C24—C23	−68.5 (2)	C6—C5—C4—C3	−179.61 (18)
C25—C5—C4—C3	−0.2 (3)	C6—C7—C8—C9	−175.93 (18)
C25—C5—C6—C7	72.9 (2)	C12—C13—C14—C15	179.61 (18)
C2—C1—C25—O1	175.13 (17)	C12—C11—C10—C9	173.74 (18)
C2—C1—C25—C5	−2.4 (3)	C8—C7—C6—C5	64.4 (2)
C2—C1—C24—C23	115.69 (19)	C8—C9—C10—C11	−1.7 (3)
C23—C28—C19—C20	−4.4 (3)	C24—C1—C25—O1	−0.8 (3)
C23—C28—C19—C18	174.25 (17)	C24—C1—C25—C5	−178.33 (18)
C23—C22—C21—C20	−2.2 (3)	C24—C1—C2—C3	177.25 (17)
C26—O2—C29—C30	74.3 (2)	C24—C23—C28—O4	2.7 (3)
C26—C7—C6—C5	−111.9 (2)	C24—C23—C28—C19	−174.19 (17)
C26—C7—C8—C9	0.5 (3)	C24—C23—C22—C21	177.62 (17)
C26—C11—C12—C13	101.7 (2)	C29—O2—C26—C7	−105.57 (19)
C26—C11—C10—C9	−1.1 (3)	C29—O2—C26—C11	79.8 (2)
C5—C4—C3—Br1	178.16 (15)	C21—C20—C19—C28	0.3 (3)
C5—C4—C3—C2	−1.1 (3)	C21—C20—C19—C18	−178.34 (17)
C28—O4—C32—C33	−170.24 (16)	C14—C13—C12—C11	108.9 (2)
C28—C23—C22—C21	−1.6 (3)	C15—C16—C17—C27	−1.3 (3)
C28—C23—C24—C1	92.9 (2)	C15—C16—C17—C18	179.50 (18)
C28—C19—C18—C17	−104.1 (2)	C17—C27—C13—C12	179.24 (17)
C4—C5—C6—C7	−107.7 (2)	C17—C27—C13—C14	−0.6 (3)
C20—C19—C18—C17	74.5 (2)	C17—C16—C15—Br3	−163.2 (8)
C7—C26—C11—C12	−170.94 (17)	C17—C16—C15—C14	0.2 (3)
C7—C26—C11—C10	3.8 (3)	C17—C16—C15—Br2	177.39 (15)
C7—C8—C9—C10	2.0 (3)	C10—C11—C12—C13	−72.9 (2)

### 5,17-Dibromo-26,28-dihydroxy-25,27-dipropynyloxycalix[4]arene (1) Hydrogen-bond geometry (Å, º)

**Table:**

*D*—H···*A*	*D*—H	H···*A*	*D*···*A*	*D*—H···*A*
O1—H1···O4	0.84	1.81	2.6270 (18)	165
O3—H3···O2	0.84	2.02	2.8207 (18)	160

### 5,17-Dibromo-26,28-dipropoxy-25,27-dipropynyloxycalix[4]arene (2) Crystal data

**Table:**

C_40_H_38_Br_2_O_4_	*D*_x_ = 1.491 Mg m^−^^3^
*M**_r_* = 742.52	Mo *K*α radiation, λ = 0.71073 Å
Orthorhombic, *P**b**c**n*	Cell parameters from 9929 reflections
*a* = 18.1223 (7) Å	θ = 2.3–31.5°
*b* = 9.9840 (4) Å	µ = 2.49 mm^−^^1^
*c* = 18.2863 (7) Å	*T* = 100 K
*V* = 3308.6 (2) Å^3^	Block, colourless
*Z* = 4	0.35 × 0.25 × 0.22 mm
*F*(000) = 1520	

### 5,17-Dibromo-26,28-dipropoxy-25,27-dipropynyloxycalix[4]arene (2) Data collection

**Table:**

Bruker D8 Venture diffractometer	5529 independent reflections
Radiation source: microfocus sealed X-ray tube, Incoatec IµS microsource	4481 reflections with *I* > 2σ(*I*)
Focusing mirrors monochromator	*R*_int_ = 0.060
Detector resolution: 10.4 pixels mm^-1^	θ_max_ = 31.5°, θ_min_ = 2.2°
ω–scan	*h* = −26→26
Absorption correction: multi-scan (*SADABS*; Krause *et al.*, 2015)	*k* = −14→14
*T*_min_ = 0.545, *T*_max_ = 0.746	*l* = −26→26
55960 measured reflections	

### 5,17-Dibromo-26,28-dipropoxy-25,27-dipropynyloxycalix[4]arene (2) Refinement

**Table:**

Refinement on *F*^2^	Primary atom site location: dual
Least-squares matrix: full	Hydrogen site location: difference Fourier map
*R*[*F*^2^ > 2σ(*F*^2^)] = 0.030	Only H-atom coordinates refined
*wR*(*F*^2^) = 0.069	*w* = 1/[σ^2^(*F*_o_^2^) + (0.0275*P*)^2^ + 1.7553*P*] where *P* = (*F*_o_^2^ + 2*F*_c_^2^)/3
*S* = 1.04	(Δ/σ)_max_ = 0.001
5529 reflections	Δρ_max_ = 0.42 e Å^−^^3^
283 parameters	Δρ_min_ = −0.51 e Å^−^^3^
0 restraints	

### 5,17-Dibromo-26,28-dipropoxy-25,27-dipropynyloxycalix[4]arene (2) Special details

**Table:**

Geometry. All esds (except the esd in the dihedral angle between two l.s. planes) are estimated using the full covariance matrix. The cell esds are taken into account individually in the estimation of esds in distances, angles and torsion angles; correlations between esds in cell parameters are only used when they are defined by crystal symmetry. An approximate (isotropic) treatment of cell esds is used for estimating esds involving l.s. planes.

### 5,17-Dibromo-26,28-dipropoxy-25,27-dipropynyloxycalix[4]arene (2) Fractional atomic coordinates and isotropic or equivalent isotropic displacement parameters (Å^2^)

**Table:**

	*x*	*y*	*z*	*U*_iso_*/*U*_eq_	
Br1	0.58375 (2)	0.73040 (2)	0.32024 (2)	0.01950 (5)	
O1	0.63861 (5)	0.13925 (9)	0.35764 (5)	0.01210 (18)	
O2	0.40187 (5)	0.38259 (9)	0.37445 (5)	0.01229 (19)	
C1	0.57053 (7)	0.33574 (14)	0.39089 (7)	0.0121 (2)	
C2	0.55903 (7)	0.47314 (14)	0.38265 (8)	0.0132 (2)	
H2	0.5264 (10)	0.5205 (18)	0.4150 (10)	0.022 (5)*	
C3	0.59636 (7)	0.54202 (14)	0.32816 (8)	0.0139 (3)	
C4	0.64115 (7)	0.47686 (13)	0.27830 (7)	0.0130 (2)	
H4	0.6605 (9)	0.5244 (16)	0.2376 (9)	0.014 (4)*	
C5	0.65355 (7)	0.33956 (13)	0.28585 (7)	0.0113 (2)	
C6	0.62149 (7)	0.27266 (13)	0.34495 (7)	0.0113 (2)	
C7	0.52365 (8)	0.25329 (14)	0.44288 (8)	0.0131 (2)	
H7A	0.5539 (10)	0.1879 (17)	0.4687 (9)	0.011 (4)*	
H7B	0.4995 (10)	0.3097 (18)	0.4804 (9)	0.017 (4)*	
C8	0.46578 (7)	0.17719 (14)	0.39939 (7)	0.0118 (2)	
C9	0.47238 (8)	0.03988 (14)	0.38663 (8)	0.0148 (3)	
H9	0.5125 (10)	−0.0059 (17)	0.4081 (9)	0.018 (4)*	
C10	0.42317 (8)	−0.02671 (15)	0.34115 (8)	0.0157 (3)	
H10	0.4300 (10)	−0.116 (2)	0.3325 (10)	0.019 (5)*	
C11	0.36687 (8)	0.04353 (14)	0.30659 (8)	0.0141 (3)	
H11	0.3354 (10)	0.0006 (18)	0.2722 (10)	0.020 (4)*	
C12	0.35870 (7)	0.18099 (14)	0.31759 (7)	0.0122 (2)	
C13	0.40748 (7)	0.24448 (13)	0.36548 (7)	0.0112 (2)	
C14	0.69566 (7)	0.26270 (14)	0.22740 (8)	0.0126 (2)	
H14A	0.7316 (10)	0.2045 (18)	0.2481 (10)	0.019 (5)*	
C15	0.70695 (8)	0.12828 (14)	0.39858 (8)	0.0139 (3)	
H15A	0.7029 (9)	0.1811 (17)	0.4442 (9)	0.012 (4)*	
H15B	0.7467 (10)	0.1659 (17)	0.3699 (9)	0.014 (4)*	
C16	0.72114 (8)	−0.01789 (14)	0.41520 (8)	0.0136 (3)	
H16A	0.7153 (9)	−0.0676 (17)	0.3706 (10)	0.015 (4)*	
H16B	0.7734 (11)	−0.0273 (18)	0.4308 (10)	0.022 (5)*	
C17	0.67134 (9)	−0.07477 (16)	0.47447 (8)	0.0182 (3)	
H17A	0.6817 (11)	−0.169 (2)	0.4837 (10)	0.027 (5)*	
H17B	0.6797 (12)	−0.027 (2)	0.5232 (12)	0.037 (6)*	
H17C	0.6186 (11)	−0.0630 (19)	0.4634 (10)	0.027 (5)*	
C18	0.35282 (8)	0.42342 (14)	0.43170 (8)	0.0148 (3)	
H18A	0.3726 (10)	0.4016 (17)	0.4800 (10)	0.016 (4)*	
H18B	0.3044 (10)	0.3780 (17)	0.4257 (9)	0.016 (4)*	
C19	0.34403 (8)	0.56881 (15)	0.42539 (8)	0.0167 (3)	
C20	0.33838 (9)	0.68613 (16)	0.41828 (9)	0.0227 (3)	
H14B	0.7229 (11)	0.324 (2)	0.1945 (11)	0.034*	
H20	0.3365 (13)	0.776 (2)	0.4114 (12)	0.041 (6)*	

### 5,17-Dibromo-26,28-dipropoxy-25,27-dipropynyloxycalix[4]arene (2) Atomic displacement parameters (Å^2^)

**Table:**

	*U*^11^	*U*^22^	*U*^33^	*U*^12^	*U*^13^	*U*^23^
Br1	0.02093 (8)	0.00958 (7)	0.02800 (9)	0.00170 (5)	−0.00372 (6)	−0.00016 (5)
O1	0.0104 (4)	0.0103 (4)	0.0157 (5)	0.0006 (3)	−0.0024 (4)	0.0012 (4)
O2	0.0125 (4)	0.0102 (4)	0.0142 (4)	0.0004 (3)	0.0032 (4)	−0.0016 (3)
C1	0.0094 (6)	0.0146 (6)	0.0124 (6)	−0.0005 (5)	−0.0023 (4)	−0.0006 (5)
C2	0.0112 (5)	0.0140 (6)	0.0146 (6)	0.0003 (5)	−0.0015 (5)	−0.0035 (5)
C3	0.0133 (6)	0.0098 (6)	0.0185 (7)	−0.0008 (4)	−0.0052 (5)	−0.0012 (5)
C4	0.0128 (6)	0.0124 (6)	0.0136 (6)	−0.0020 (5)	−0.0024 (5)	0.0014 (5)
C5	0.0092 (5)	0.0127 (6)	0.0121 (6)	−0.0005 (4)	−0.0021 (4)	−0.0008 (5)
C6	0.0099 (5)	0.0104 (6)	0.0136 (6)	−0.0005 (5)	−0.0027 (5)	0.0002 (5)
C7	0.0119 (6)	0.0148 (6)	0.0125 (6)	0.0001 (5)	0.0004 (5)	0.0011 (5)
C8	0.0107 (5)	0.0140 (6)	0.0107 (6)	−0.0004 (5)	0.0025 (5)	0.0007 (5)
C9	0.0131 (6)	0.0146 (6)	0.0167 (6)	0.0023 (5)	0.0017 (5)	0.0030 (5)
C10	0.0166 (6)	0.0115 (6)	0.0188 (7)	0.0002 (5)	0.0041 (5)	0.0006 (5)
C11	0.0136 (6)	0.0135 (6)	0.0152 (6)	−0.0015 (5)	0.0007 (5)	−0.0004 (5)
C12	0.0113 (6)	0.0129 (6)	0.0125 (6)	−0.0006 (5)	0.0020 (5)	0.0015 (5)
C13	0.0115 (6)	0.0111 (6)	0.0111 (6)	−0.0005 (4)	0.0033 (4)	0.0007 (4)
C14	0.0098 (6)	0.0143 (6)	0.0138 (6)	0.0007 (5)	−0.0002 (5)	0.0004 (5)
C15	0.0115 (6)	0.0144 (6)	0.0157 (6)	0.0003 (5)	−0.0028 (5)	0.0018 (5)
C16	0.0124 (6)	0.0137 (6)	0.0148 (6)	0.0028 (5)	0.0017 (5)	0.0030 (5)
C17	0.0169 (7)	0.0198 (7)	0.0181 (7)	0.0017 (6)	0.0015 (5)	0.0065 (6)
C18	0.0165 (6)	0.0138 (6)	0.0141 (6)	0.0004 (5)	0.0044 (5)	−0.0019 (5)
C19	0.0151 (6)	0.0194 (7)	0.0156 (6)	0.0006 (5)	0.0029 (5)	−0.0023 (5)
C20	0.0268 (8)	0.0173 (7)	0.0240 (8)	0.0014 (6)	0.0063 (6)	−0.0012 (6)

### 5,17-Dibromo-26,28-dipropoxy-25,27-dipropynyloxycalix[4]arene (2) Geometric parameters (Å, º)

**Table:**

Br1—C3	1.9002 (14)	C10—H10	0.91 (2)
O1—C6	1.3872 (16)	C10—C11	1.390 (2)
O1—C15	1.4513 (16)	C11—H11	0.950 (18)
O2—C13	1.3923 (15)	C11—C12	1.3949 (19)
O2—C18	1.4325 (16)	C12—C13	1.3966 (19)
C1—C2	1.3957 (19)	C12—C14^i^	1.5209 (19)
C1—C6	1.3982 (18)	C14—H14A	0.952 (18)
C1—C7	1.5176 (19)	C14—H14B	0.99 (2)
C2—H2	0.961 (18)	C15—H15A	0.990 (17)
C2—C3	1.387 (2)	C15—H15B	0.967 (17)
C3—C4	1.3833 (19)	C15—C16	1.5126 (19)
C4—H4	0.950 (17)	C16—H16A	0.962 (18)
C4—C5	1.3959 (18)	C16—H16B	0.992 (19)
C5—C6	1.3971 (19)	C16—C17	1.520 (2)
C5—C14	1.5211 (19)	C17—H17A	0.97 (2)
C7—H7A	0.974 (17)	C17—H17B	1.02 (2)
C7—H7B	0.990 (18)	C17—H17C	0.98 (2)
C7—C8	1.5197 (19)	C18—H18A	0.978 (17)
C8—C9	1.3957 (19)	C18—H18B	0.993 (18)
C8—C13	1.3971 (18)	C18—C19	1.465 (2)
C9—H9	0.944 (18)	C19—C20	1.183 (2)
C9—C10	1.389 (2)	C20—H20	0.90 (2)
			
C6—O1—C15	110.46 (10)	C11—C12—C13	118.02 (12)
C13—O2—C18	114.41 (10)	C11—C12—C14^i^	121.23 (12)
C2—C1—C6	118.46 (12)	C13—C12—C14^i^	120.38 (12)
C2—C1—C7	121.14 (12)	O2—C13—C8	118.64 (12)
C6—C1—C7	120.12 (12)	O2—C13—C12	118.50 (12)
C1—C2—H2	120.6 (11)	C12—C13—C8	122.59 (12)
C3—C2—C1	119.48 (13)	C5—C14—H14A	111.9 (11)
C3—C2—H2	119.9 (11)	C5—C14—H14B	111.3 (13)
C2—C3—Br1	119.14 (10)	C12^i^—C14—C5	109.01 (11)
C4—C3—Br1	119.07 (11)	C12^i^—C14—H14A	109.3 (11)
C4—C3—C2	121.77 (13)	C12^i^—C14—H14B	109.0 (12)
C3—C4—H4	119.9 (10)	H14A—C14—H14B	106.2 (16)
C3—C4—C5	119.42 (13)	O1—C15—H15A	109.4 (10)
C5—C4—H4	120.6 (10)	O1—C15—H15B	109.1 (10)
C4—C5—C6	118.62 (12)	O1—C15—C16	108.76 (11)
C4—C5—C14	120.45 (12)	H15A—C15—H15B	107.8 (14)
C6—C5—C14	120.73 (12)	C16—C15—H15A	110.9 (10)
O1—C6—C1	118.67 (12)	C16—C15—H15B	110.9 (10)
O1—C6—C5	119.70 (12)	C15—C16—H16A	108.0 (10)
C5—C6—C1	121.60 (12)	C15—C16—H16B	108.1 (10)
C1—C7—H7A	110.6 (10)	C15—C16—C17	113.74 (12)
C1—C7—H7B	111.9 (10)	H16A—C16—H16B	107.4 (14)
C1—C7—C8	109.25 (11)	C17—C16—H16A	110.3 (10)
H7A—C7—H7B	107.1 (14)	C17—C16—H16B	109.0 (10)
C8—C7—H7A	107.9 (10)	C16—C17—H17A	111.7 (11)
C8—C7—H7B	110.0 (10)	C16—C17—H17B	111.1 (12)
C9—C8—C7	121.29 (12)	C16—C17—H17C	112.6 (11)
C9—C8—C13	117.57 (12)	H17A—C17—H17B	105.8 (16)
C13—C8—C7	120.91 (12)	H17A—C17—H17C	109.8 (16)
C8—C9—H9	118.1 (11)	H17B—C17—H17C	105.5 (16)
C10—C9—C8	121.02 (13)	O2—C18—H18A	111.7 (10)
C10—C9—H9	120.8 (11)	O2—C18—H18B	109.8 (10)
C9—C10—H10	118.9 (12)	O2—C18—C19	106.97 (11)
C9—C10—C11	120.13 (13)	H18A—C18—H18B	108.7 (14)
C11—C10—H10	120.8 (12)	C19—C18—H18A	109.4 (10)
C10—C11—H11	120.9 (11)	C19—C18—H18B	110.3 (10)
C10—C11—C12	120.58 (13)	C20—C19—C18	177.81 (16)
C12—C11—H11	118.4 (11)	C19—C20—H20	176.8 (15)
			
Br1—C3—C4—C5	176.93 (10)	C7—C8—C9—C10	−173.85 (13)
O1—C15—C16—C17	−73.91 (15)	C7—C8—C13—O2	−2.26 (18)
C1—C2—C3—Br1	−177.17 (10)	C7—C8—C13—C12	171.66 (12)
C1—C2—C3—C4	4.4 (2)	C8—C9—C10—C11	1.0 (2)
C1—C7—C8—C9	105.00 (14)	C9—C8—C13—O2	−176.87 (12)
C1—C7—C8—C13	−69.41 (16)	C9—C8—C13—C12	−2.95 (19)
C2—C1—C6—O1	173.62 (12)	C9—C10—C11—C12	−0.6 (2)
C2—C1—C6—C5	−8.25 (19)	C10—C11—C12—C13	−1.5 (2)
C2—C1—C7—C8	101.52 (14)	C10—C11—C12—C14^i^	171.60 (13)
C2—C3—C4—C5	−4.7 (2)	C11—C12—C13—O2	177.27 (11)
C3—C4—C5—C6	−1.51 (19)	C11—C12—C13—C8	3.34 (19)
C3—C4—C5—C14	173.39 (12)	C13—O2—C18—C19	−170.66 (11)
C4—C5—C6—O1	−173.87 (11)	C13—C8—C9—C10	0.7 (2)
C4—C5—C6—C1	8.02 (19)	C14—C5—C6—O1	11.25 (19)
C4—C5—C14—C12^i^	−104.49 (14)	C14—C5—C6—C1	−166.87 (12)
C6—O1—C15—C16	176.59 (11)	C14^i^—C12—C13—O2	4.09 (18)
C6—C1—C2—C3	1.98 (19)	C14^i^—C12—C13—C8	−169.84 (12)
C6—C1—C7—C8	−72.24 (15)	C15—O1—C6—C1	−98.40 (14)
C6—C5—C14—C12^i^	70.31 (16)	C15—O1—C6—C5	83.44 (15)
C7—C1—C2—C3	−171.87 (12)	C18—O2—C13—C8	−96.01 (14)
C7—C1—C6—O1	−12.46 (18)	C18—O2—C13—C12	89.82 (14)
C7—C1—C6—C5	165.67 (12)		

Symmetry code: (i) −*x*+1, *y*, −*z*+1/2.

### 25,27-Bis(2-azidoethoxy)-5,17-dibromo-26,28-dihydroxycalix[4]arene (3) Crystal data

**Table:**

C_32_H_28_Br_2_N_6_O_4_	*D*_x_ = 1.557 Mg m^−^^3^
*M**_r_* = 720.42	Mo *K*α radiation, λ = 0.71073 Å
Trigonal, *R*3	Cell parameters from 9927 reflections
*a* = 36.3261 (7) Å	θ = 2.2–28.3°
*c* = 12.1054 (4) Å	µ = 2.69 mm^−^^1^
*V* = 13834.0 (7) Å^3^	*T* = 100 K
*Z* = 18	Block, colourless
*F*(000) = 6552	0.37 × 0.13 × 0.1 mm

### 25,27-Bis(2-azidoethoxy)-5,17-dibromo-26,28-dihydroxycalix[4]arene (3) Data collection

**Table:**

Bruker D8 Venture diffractometer	7804 independent reflections
Radiation source: microfocus sealed X-ray tube, Incoatec IµS microsource	6043 reflections with *I* > 2σ(*I*)
Focusing mirrors monochromator	*R*_int_ = 0.047
Detector resolution: 10.4 pixels mm^-1^	θ_max_ = 28.5°, θ_min_ = 1.8°
ω–scan	*h* = −47→48
Absorption correction: multi-scan (*SADABS*; Krause *et al.*, 2015)	*k* = −48→48
*T*_min_ = 0.516, *T*_max_ = 0.746	*l* = −16→16
45251 measured reflections	

### 25,27-Bis(2-azidoethoxy)-5,17-dibromo-26,28-dihydroxycalix[4]arene (3) Refinement

**Table:**

Refinement on *F*^2^	24 restraints
Least-squares matrix: full	Hydrogen site location: inferred from neighbouring sites
*R*[*F*^2^ > 2σ(*F*^2^)] = 0.040	H-atom parameters constrained
*wR*(*F*^2^) = 0.097	*w* = 1/[σ^2^(*F*_o_^2^) + (0.0383*P*)^2^ + 55.0341*P*] where *P* = (*F*_o_^2^ + 2*F*_c_^2^)/3
*S* = 1.03	(Δ/σ)_max_ = 0.003
7804 reflections	Δρ_max_ = 1.30 e Å^−^^3^
399 parameters	Δρ_min_ = −1.12 e Å^−^^3^

### 25,27-Bis(2-azidoethoxy)-5,17-dibromo-26,28-dihydroxycalix[4]arene (3) Special details

**Table:**

Geometry. All esds (except the esd in the dihedral angle between two l.s. planes) are estimated using the full covariance matrix. The cell esds are taken into account individually in the estimation of esds in distances, angles and torsion angles; correlations between esds in cell parameters are only used when they are defined by crystal symmetry. An approximate (isotropic) treatment of cell esds is used for estimating esds involving l.s. planes.

### 25,27-Bis(2-azidoethoxy)-5,17-dibromo-26,28-dihydroxycalix[4]arene (3) Fractional atomic coordinates and isotropic or equivalent isotropic displacement parameters (Å^2^)

**Table:**

	*x*	*y*	*z*	*U*_iso_*/*U*_eq_	
Br2	0.77808 (2)	0.64422 (2)	0.11845 (2)	0.02592 (8)	
Br1	0.94747 (2)	0.64087 (2)	1.04746 (3)	0.03614 (9)	
O4	0.73344 (6)	0.53322 (6)	0.69360 (15)	0.0235 (4)	
O3	0.76702 (7)	0.53195 (7)	0.49646 (16)	0.0304 (5)	
H3	0.754079	0.534255	0.551158	0.046*	
O1	0.81215 (6)	0.53626 (7)	0.71561 (16)	0.0296 (4)	
H1	0.821251	0.526224	0.667258	0.044*	
O2	0.82909 (7)	0.50491 (6)	0.52611 (17)	0.0317 (5)	
C16	0.75008 (8)	0.60698 (9)	0.3311 (2)	0.0237 (5)	
H16	0.735357	0.622450	0.332302	0.028*	
C15	0.77434 (8)	0.60962 (9)	0.2407 (2)	0.0233 (5)	
C3	0.90557 (9)	0.61047 (9)	0.9377 (2)	0.0260 (6)	
C2	0.86500 (9)	0.60483 (9)	0.9485 (2)	0.0241 (5)	
H2	0.858609	0.617607	1.008329	0.029*	
C24	0.78870 (8)	0.57243 (9)	0.8817 (2)	0.0231 (5)	
H24A	0.784545	0.580534	0.956822	0.028*	
H24B	0.768569	0.541658	0.872265	0.028*	
C10	0.89056 (9)	0.60072 (9)	0.3603 (2)	0.0274 (6)	
H10	0.889258	0.612151	0.290757	0.033*	
C20	0.76455 (9)	0.64427 (10)	0.6366 (2)	0.0296 (6)	
H20	0.760226	0.660610	0.582331	0.036*	
C17	0.74717 (8)	0.58147 (9)	0.4209 (2)	0.0241 (5)	
C11	0.85771 (9)	0.56095 (9)	0.3911 (2)	0.0255 (6)	
C14	0.79580 (8)	0.58721 (9)	0.2352 (2)	0.0238 (5)	
H14	0.812203	0.589504	0.171789	0.029*	
C19	0.74568 (9)	0.60024 (9)	0.6231 (2)	0.0257 (6)	
C25	0.84464 (9)	0.56262 (9)	0.7832 (2)	0.0248 (6)	
C28	0.75217 (8)	0.57733 (9)	0.7062 (2)	0.0226 (5)	
C13	0.79333 (8)	0.56136 (9)	0.3226 (2)	0.0241 (5)	
C8	0.92731 (9)	0.60764 (10)	0.5302 (2)	0.0308 (6)	
H8	0.950790	0.624029	0.577501	0.037*	
C1	0.83381 (9)	0.58053 (9)	0.8717 (2)	0.0231 (5)	
C5	0.88617 (9)	0.56989 (9)	0.7704 (2)	0.0277 (6)	
C23	0.77852 (8)	0.59706 (9)	0.7970 (2)	0.0227 (5)	
C22	0.79695 (9)	0.64103 (9)	0.8067 (2)	0.0274 (6)	
H22	0.814878	0.655309	0.867784	0.033*	
C21	0.78926 (10)	0.66417 (10)	0.7275 (2)	0.0304 (6)	
H21	0.801203	0.693985	0.736316	0.037*	
C7	0.89577 (9)	0.56776 (10)	0.5635 (2)	0.0277 (6)	
C4	0.91627 (9)	0.59369 (10)	0.8493 (2)	0.0296 (6)	
H4	0.944407	0.598510	0.842644	0.036*	
C26	0.86166 (9)	0.54477 (9)	0.4921 (2)	0.0261 (6)	
C27	0.76893 (9)	0.55853 (9)	0.4151 (2)	0.0248 (6)	
C18	0.72006 (9)	0.57839 (10)	0.5199 (2)	0.0287 (6)	
H18A	0.700519	0.548016	0.536836	0.034*	
H18B	0.702496	0.591249	0.500219	0.034*	
C31	0.69060 (9)	0.50925 (10)	0.7339 (2)	0.0309 (6)	
H31A	0.674011	0.522648	0.709611	0.037*	
H31B	0.690578	0.508623	0.815710	0.037*	
C9	0.92504 (9)	0.62399 (10)	0.4281 (3)	0.0316 (6)	
H9	0.947183	0.651128	0.405307	0.038*	
N4	0.66469 (11)	0.46548 (11)	0.5687 (3)	0.0530 (8)	
C6	0.89773 (11)	0.55028 (11)	0.6759 (2)	0.0337 (7)	
H6A	0.878111	0.519168	0.676259	0.040*	
H6B	0.926863	0.555358	0.687975	0.040*	
C12	0.81799 (9)	0.53781 (10)	0.3211 (2)	0.0293 (6)	
H12A	0.799872	0.508657	0.350041	0.035*	
H12B	0.825950	0.535615	0.244215	0.035*	
C32	0.67100 (10)	0.46485 (11)	0.6891 (3)	0.0393 (7)	
H32A	0.689825	0.453040	0.704496	0.047*	
H32B	0.643333	0.446505	0.725666	0.047*	
C29	0.83526 (12)	0.47048 (10)	0.4902 (3)	0.0410 (8)	
H29A	0.860714	0.472574	0.526160	0.049*	
H29B	0.839456	0.471768	0.409152	0.049*	
N5	0.68006 (13)	0.44967 (14)	0.5103 (3)	0.0736 (11)	
C30	0.79672 (14)	0.42988 (11)	0.5213 (4)	0.0551 (10)	
H30A	0.797717	0.405718	0.486638	0.066*	
H30B	0.770974	0.429853	0.494378	0.066*	
N6	0.69206 (17)	0.43548 (18)	0.4454 (4)	0.1068 (16)	
N1	0.79469 (12)	0.42496 (12)	0.6448 (3)	0.0627 (10)	
N2	0.76567 (14)	0.42481 (14)	0.6888 (3)	0.0696 (10)	
N3	0.74014 (17)	0.42453 (19)	0.7425 (4)	0.0991 (13)	

### 25,27-Bis(2-azidoethoxy)-5,17-dibromo-26,28-dihydroxycalix[4]arene (3) Atomic displacement parameters (Å^2^)

**Table:**

	*U*^11^	*U*^22^	*U*^33^	*U*^12^	*U*^13^	*U*^23^
Br2	0.02212 (13)	0.02979 (15)	0.02155 (13)	0.00975 (11)	−0.00145 (10)	0.00350 (11)
Br1	0.02894 (16)	0.03163 (16)	0.04687 (19)	0.01439 (13)	−0.00821 (13)	−0.00860 (13)
O4	0.0183 (9)	0.0272 (10)	0.0253 (9)	0.0116 (8)	0.0057 (7)	0.0035 (8)
O3	0.0372 (12)	0.0398 (12)	0.0249 (10)	0.0273 (10)	0.0095 (9)	0.0086 (9)
O1	0.0292 (10)	0.0423 (12)	0.0267 (10)	0.0249 (10)	−0.0010 (8)	−0.0068 (9)
O2	0.0373 (11)	0.0288 (11)	0.0378 (12)	0.0233 (10)	0.0125 (9)	0.0051 (9)
C16	0.0219 (13)	0.0267 (14)	0.0244 (13)	0.0135 (11)	−0.0011 (10)	0.0006 (11)
C15	0.0197 (12)	0.0256 (13)	0.0197 (12)	0.0077 (11)	−0.0018 (10)	0.0023 (10)
C3	0.0247 (14)	0.0212 (13)	0.0298 (14)	0.0098 (11)	−0.0020 (11)	0.0003 (11)
C2	0.0282 (14)	0.0241 (13)	0.0225 (13)	0.0150 (12)	0.0035 (11)	0.0017 (10)
C24	0.0244 (13)	0.0294 (14)	0.0189 (12)	0.0160 (12)	0.0042 (10)	0.0027 (10)
C10	0.0304 (15)	0.0353 (15)	0.0268 (14)	0.0241 (13)	0.0082 (11)	0.0067 (12)
C20	0.0309 (15)	0.0396 (16)	0.0299 (15)	0.0263 (14)	0.0116 (12)	0.0124 (13)
C17	0.0216 (13)	0.0302 (14)	0.0225 (13)	0.0144 (11)	0.0015 (10)	0.0029 (11)
C11	0.0276 (14)	0.0314 (14)	0.0276 (14)	0.0223 (12)	0.0028 (11)	−0.0030 (11)
C14	0.0196 (12)	0.0277 (14)	0.0208 (12)	0.0093 (11)	0.0007 (10)	−0.0030 (11)
C19	0.0247 (13)	0.0369 (15)	0.0236 (13)	0.0215 (12)	0.0088 (11)	0.0069 (11)
C25	0.0297 (14)	0.0289 (14)	0.0213 (13)	0.0189 (12)	0.0016 (11)	0.0031 (11)
C28	0.0214 (12)	0.0284 (14)	0.0236 (13)	0.0167 (11)	0.0084 (10)	0.0041 (11)
C13	0.0224 (13)	0.0267 (14)	0.0240 (13)	0.0128 (11)	−0.0013 (10)	−0.0031 (11)
C8	0.0274 (15)	0.0386 (17)	0.0346 (16)	0.0227 (14)	−0.0008 (12)	−0.0072 (13)
C1	0.0246 (13)	0.0256 (13)	0.0238 (13)	0.0161 (11)	0.0054 (11)	0.0046 (11)
C5	0.0356 (16)	0.0351 (15)	0.0246 (13)	0.0268 (13)	0.0064 (12)	0.0052 (12)
C23	0.0223 (13)	0.0302 (14)	0.0214 (12)	0.0174 (11)	0.0077 (10)	0.0045 (11)
C22	0.0284 (14)	0.0332 (15)	0.0257 (14)	0.0192 (13)	0.0083 (11)	0.0015 (11)
C21	0.0341 (16)	0.0288 (15)	0.0352 (16)	0.0208 (13)	0.0124 (13)	0.0047 (12)
C7	0.0306 (15)	0.0393 (16)	0.0278 (14)	0.0283 (14)	0.0034 (11)	−0.0011 (12)
C4	0.0257 (14)	0.0385 (16)	0.0307 (15)	0.0205 (13)	0.0059 (12)	0.0066 (13)
C26	0.0299 (14)	0.0278 (14)	0.0292 (14)	0.0209 (12)	0.0084 (11)	0.0024 (11)
C27	0.0229 (13)	0.0281 (14)	0.0244 (13)	0.0134 (11)	0.0006 (10)	0.0033 (11)
C18	0.0239 (14)	0.0434 (17)	0.0262 (14)	0.0224 (13)	0.0051 (11)	0.0097 (12)
C31	0.0204 (13)	0.0369 (16)	0.0323 (15)	0.0118 (12)	0.0079 (11)	0.0106 (13)
C9	0.0260 (14)	0.0332 (16)	0.0408 (17)	0.0188 (13)	0.0081 (13)	0.0022 (13)
N4	0.0470 (18)	0.0516 (19)	0.0449 (18)	0.0130 (15)	−0.0156 (15)	−0.0026 (15)
C6	0.0432 (18)	0.0516 (19)	0.0262 (15)	0.0386 (16)	0.0029 (13)	0.0018 (13)
C12	0.0338 (15)	0.0337 (15)	0.0267 (14)	0.0215 (13)	0.0005 (12)	−0.0049 (12)
C32	0.0264 (15)	0.0382 (18)	0.0444 (19)	0.0094 (14)	−0.0015 (14)	0.0092 (15)
C29	0.055 (2)	0.0330 (17)	0.0484 (19)	0.0324 (17)	0.0147 (16)	0.0051 (14)
N5	0.066 (2)	0.071 (2)	0.059 (2)	0.0148 (19)	−0.0020 (19)	−0.0098 (19)
C30	0.067 (3)	0.0323 (18)	0.072 (3)	0.0296 (19)	0.008 (2)	0.0039 (18)
N6	0.103 (3)	0.106 (3)	0.080 (3)	0.029 (3)	0.016 (3)	−0.020 (3)
N1	0.066 (2)	0.063 (2)	0.074 (2)	0.0436 (19)	0.0163 (19)	0.0304 (19)
N2	0.075 (2)	0.096 (3)	0.063 (2)	0.063 (2)	−0.0062 (19)	−0.004 (2)
N3	0.098 (3)	0.151 (4)	0.074 (3)	0.082 (3)	−0.008 (2)	−0.011 (3)

### 25,27-Bis(2-azidoethoxy)-5,17-dibromo-26,28-dihydroxycalix[4]arene (3) Geometric parameters (Å, º)

**Table:**

Br2—C15	1.902 (3)	C28—C23	1.397 (4)
Br1—C3	1.903 (3)	C13—C27	1.400 (4)
O4—C28	1.401 (3)	C13—C12	1.517 (4)
O4—C31	1.436 (3)	C8—H8	0.9500
O3—H3	0.8400	C8—C7	1.383 (4)
O3—C27	1.356 (3)	C8—C9	1.391 (4)
O1—H1	0.8400	C5—C4	1.382 (4)
O1—C25	1.360 (3)	C5—C6	1.514 (4)
O2—C26	1.397 (3)	C23—C22	1.394 (4)
O2—C29	1.444 (3)	C22—H22	0.9500
C16—H16	0.9500	C22—C21	1.392 (4)
C16—C15	1.377 (4)	C21—H21	0.9500
C16—C17	1.399 (4)	C7—C26	1.394 (4)
C15—C14	1.382 (4)	C7—C6	1.519 (4)
C3—C2	1.389 (4)	C4—H4	0.9500
C3—C4	1.380 (4)	C18—H18A	0.9900
C2—H2	0.9500	C18—H18B	0.9900
C2—C1	1.388 (4)	C31—H31A	0.9900
C24—H24A	0.9900	C31—H31B	0.9900
C24—H24B	0.9900	C31—C32	1.501 (5)
C24—C1	1.518 (4)	C9—H9	0.9500
C24—C23	1.523 (4)	N4—C32	1.477 (4)
C10—H10	0.9500	N4—N5	1.209 (5)
C10—C11	1.388 (4)	C6—H6A	0.9900
C10—C9	1.378 (4)	C6—H6B	0.9900
C20—H20	0.9500	C12—H12A	0.9900
C20—C19	1.400 (4)	C12—H12B	0.9900
C20—C21	1.374 (4)	C32—H32A	0.9900
C17—C27	1.408 (4)	C32—H32B	0.9900
C17—C18	1.519 (4)	C29—H29A	0.9900
C11—C26	1.394 (4)	C29—H29B	0.9900
C11—C12	1.515 (4)	C29—C30	1.488 (5)
C14—H14	0.9500	N5—N6	1.139 (6)
C14—C13	1.387 (4)	C30—H30A	0.9900
C19—C28	1.399 (4)	C30—H30B	0.9900
C19—C18	1.522 (4)	C30—N1	1.502 (5)
C25—C1	1.408 (4)	N1—N2	1.179 (5)
C25—C5	1.404 (4)	N2—N3	1.128 (6)
			
C28—O4—C31	114.6 (2)	C20—C21—C22	120.6 (3)
C27—O3—H3	109.5	C20—C21—H21	119.7
C25—O1—H1	109.5	C22—C21—H21	119.7
C26—O2—C29	113.0 (2)	C8—C7—C26	118.0 (3)
C15—C16—H16	120.1	C8—C7—C6	120.8 (3)
C15—C16—C17	119.8 (2)	C26—C7—C6	121.2 (3)
C17—C16—H16	120.1	C3—C4—C5	120.2 (3)
C16—C15—Br2	119.7 (2)	C3—C4—H4	119.9
C16—C15—C14	121.8 (2)	C5—C4—H4	119.9
C14—C15—Br2	118.5 (2)	C11—C26—O2	118.9 (3)
C2—C3—Br1	120.1 (2)	C7—C26—O2	118.5 (3)
C4—C3—Br1	118.5 (2)	C7—C26—C11	122.6 (3)
C4—C3—C2	121.3 (3)	O3—C27—C17	122.8 (2)
C3—C2—H2	120.0	O3—C27—C13	116.0 (2)
C1—C2—C3	120.0 (3)	C13—C27—C17	121.2 (2)
C1—C2—H2	120.0	C17—C18—C19	113.8 (2)
H24A—C24—H24B	107.8	C17—C18—H18A	108.8
C1—C24—H24A	109.0	C17—C18—H18B	108.8
C1—C24—H24B	109.0	C19—C18—H18A	108.8
C1—C24—C23	112.8 (2)	C19—C18—H18B	108.8
C23—C24—H24A	109.0	H18A—C18—H18B	107.7
C23—C24—H24B	109.0	O4—C31—H31A	110.0
C11—C10—H10	119.2	O4—C31—H31B	110.0
C9—C10—H10	119.2	O4—C31—C32	108.4 (2)
C9—C10—C11	121.5 (3)	H31A—C31—H31B	108.4
C19—C20—H20	119.6	C32—C31—H31A	110.0
C21—C20—H20	119.6	C32—C31—H31B	110.0
C21—C20—C19	120.8 (3)	C10—C9—C8	119.8 (3)
C16—C17—C27	118.3 (2)	C10—C9—H9	120.1
C16—C17—C18	119.6 (2)	C8—C9—H9	120.1
C27—C17—C18	122.1 (2)	N5—N4—C32	117.2 (4)
C10—C11—C26	117.3 (3)	C5—C6—C7	113.5 (2)
C10—C11—C12	120.8 (3)	C5—C6—H6A	108.9
C26—C11—C12	121.8 (3)	C5—C6—H6B	108.9
C15—C14—H14	120.1	C7—C6—H6A	108.9
C15—C14—C13	119.9 (2)	C7—C6—H6B	108.9
C13—C14—H14	120.1	H6A—C6—H6B	107.7
C20—C19—C18	120.9 (2)	C11—C12—C13	110.4 (2)
C28—C19—C20	117.6 (3)	C11—C12—H12A	109.6
C28—C19—C18	121.4 (3)	C11—C12—H12B	109.6
O1—C25—C1	116.0 (2)	C13—C12—H12A	109.6
O1—C25—C5	122.6 (2)	C13—C12—H12B	109.6
C5—C25—C1	121.3 (3)	H12A—C12—H12B	108.1
C19—C28—O4	118.2 (2)	C31—C32—H32A	109.8
C23—C28—O4	119.3 (2)	C31—C32—H32B	109.8
C23—C28—C19	122.4 (3)	N4—C32—C31	109.5 (3)
C14—C13—C27	118.9 (2)	N4—C32—H32A	109.8
C14—C13—C12	121.1 (2)	N4—C32—H32B	109.8
C27—C13—C12	119.9 (2)	H32A—C32—H32B	108.2
C7—C8—H8	119.6	O2—C29—H29A	110.1
C7—C8—C9	120.7 (3)	O2—C29—H29B	110.1
C9—C8—H8	119.6	O2—C29—C30	107.8 (3)
C2—C1—C24	121.9 (2)	H29A—C29—H29B	108.5
C2—C1—C25	118.4 (2)	C30—C29—H29A	110.1
C25—C1—C24	119.7 (2)	C30—C29—H29B	110.1
C25—C5—C6	121.3 (3)	N6—N5—N4	172.0 (6)
C4—C5—C25	118.8 (3)	C29—C30—H30A	109.7
C4—C5—C6	119.8 (3)	C29—C30—H30B	109.7
C28—C23—C24	122.3 (2)	C29—C30—N1	109.6 (3)
C22—C23—C24	119.8 (3)	H30A—C30—H30B	108.2
C22—C23—C28	117.9 (2)	N1—C30—H30A	109.7
C23—C22—H22	119.8	N1—C30—H30B	109.7
C21—C22—C23	120.5 (3)	N2—N1—C30	116.2 (4)
C21—C22—H22	119.8	N3—N2—N1	171.8 (5)
			
Br2—C15—C14—C13	−179.3 (2)	C8—C7—C26—O2	−178.4 (2)
Br1—C3—C2—C1	−176.2 (2)	C8—C7—C26—C11	−2.0 (4)
Br1—C3—C4—C5	177.3 (2)	C8—C7—C6—C5	75.5 (4)
O4—C28—C23—C24	1.7 (4)	C1—C24—C23—C28	106.9 (3)
O4—C28—C23—C22	179.5 (2)	C1—C24—C23—C22	−70.9 (3)
O4—C31—C32—N4	−69.0 (3)	C1—C25—C5—C4	2.7 (4)
O1—C25—C1—C2	174.8 (2)	C1—C25—C5—C6	179.4 (3)
O1—C25—C1—C24	−4.4 (4)	C5—C25—C1—C2	−1.7 (4)
O1—C25—C5—C4	−173.5 (3)	C5—C25—C1—C24	179.1 (2)
O1—C25—C5—C6	3.1 (4)	C23—C24—C1—C2	107.3 (3)
O2—C29—C30—N1	−69.2 (4)	C23—C24—C1—C25	−73.5 (3)
C16—C15—C14—C13	−0.3 (4)	C23—C22—C21—C20	−2.2 (4)
C16—C17—C27—O3	−177.9 (3)	C21—C20—C19—C28	1.3 (4)
C16—C17—C27—C13	0.7 (4)	C21—C20—C19—C18	−177.0 (2)
C16—C17—C18—C19	−109.8 (3)	C7—C8—C9—C10	1.5 (4)
C15—C16—C17—C27	−1.0 (4)	C4—C3—C2—C1	2.5 (4)
C15—C16—C17—C18	−179.5 (3)	C4—C5—C6—C7	−115.9 (3)
C15—C14—C13—C27	0.0 (4)	C26—O2—C29—C30	−174.4 (3)
C15—C14—C13—C12	−177.4 (2)	C26—C11—C12—C13	108.9 (3)
C3—C2—C1—C24	178.3 (2)	C26—C7—C6—C5	−102.6 (3)
C3—C2—C1—C25	−0.9 (4)	C27—C17—C18—C19	71.7 (4)
C2—C3—C4—C5	−1.5 (4)	C27—C13—C12—C11	−76.6 (3)
C24—C23—C22—C21	177.5 (2)	C18—C17—C27—O3	0.6 (4)
C10—C11—C26—O2	179.7 (2)	C18—C17—C27—C13	179.2 (3)
C10—C11—C26—C7	3.3 (4)	C18—C19—C28—O4	−1.6 (4)
C10—C11—C12—C13	−66.7 (3)	C18—C19—C28—C23	174.4 (2)
C20—C19—C28—O4	−180.0 (2)	C31—O4—C28—C19	−86.8 (3)
C20—C19—C28—C23	−4.0 (4)	C31—O4—C28—C23	97.1 (3)
C20—C19—C18—C17	80.6 (3)	C9—C10—C11—C26	−2.2 (4)
C17—C16—C15—Br2	179.8 (2)	C9—C10—C11—C12	173.6 (2)
C17—C16—C15—C14	0.8 (4)	C9—C8—C7—C26	−0.5 (4)
C11—C10—C9—C8	−0.1 (4)	C9—C8—C7—C6	−178.6 (2)
C14—C13—C27—O3	178.5 (2)	C6—C5—C4—C3	−177.8 (3)
C14—C13—C27—C17	−0.2 (4)	C6—C7—C26—O2	−0.2 (4)
C14—C13—C12—C11	100.8 (3)	C6—C7—C26—C11	176.2 (2)
C19—C20—C21—C22	1.7 (4)	C12—C11—C26—O2	3.9 (4)
C19—C28—C23—C24	−174.3 (2)	C12—C11—C26—C7	−172.5 (2)
C19—C28—C23—C22	3.5 (4)	C12—C13—C27—O3	−4.0 (4)
C25—C5—C4—C3	−1.1 (4)	C12—C13—C27—C17	177.2 (3)
C25—C5—C6—C7	67.5 (4)	C29—O2—C26—C11	90.4 (3)
C28—O4—C31—C32	165.3 (2)	C29—O2—C26—C7	−93.0 (3)
C28—C19—C18—C17	−97.7 (3)	C29—C30—N1—N2	115.8 (4)
C28—C23—C22—C21	−0.4 (4)	N5—N4—C32—C31	125.4 (4)

### 25,27-Bis(2-azidoethoxy)-5,17-dibromo-26,28-dihydroxycalix[4]arene (3) Hydrogen-bond geometry (Å, º)

**Table:**

*D*—H···*A*	*D*—H	H···*A*	*D*···*A*	*D*—H···*A*
O3—H3···O4	0.84	1.87	2.691 (3)	164
O1—H1···O2	0.84	1.95	2.764 (3)	162
