# Supplementary material for: Synthesis and crystal structures of 5,17-di­bromo-26,28-dihy­droxy-25,27-dipropynyloxycalix[4]arene, 5,17-di­bromo-26,28-dipropoxy-25,27-dipropynyloxycalix[4]arene and 25,27-bis­(2-azido­eth­oxy)-5,17-di­bromo-26,28-di­hydroxy­calix[4]arene
Source: Acta Crystallogr E Crystallogr Commun. 2024 May 3;80(Pt 6):555–60. doi: 10.1107/S2056989024003785 (PMC11151303; doi:10.1107/S2056989024003785)
Supplement: Supplementary file 8 [file e-80-00555-sup8.pdf]

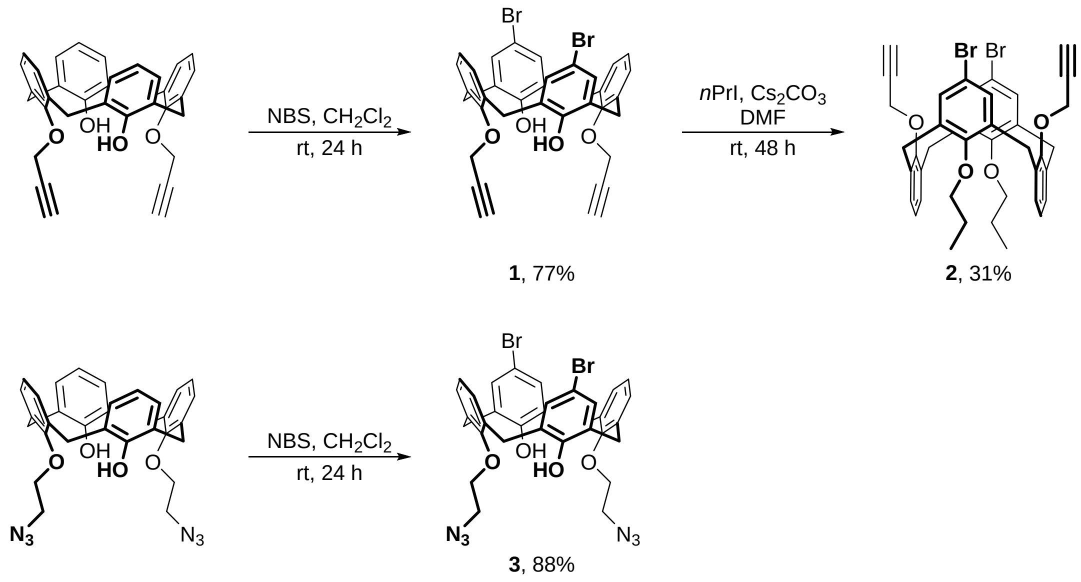

Scheme S1. Synthesis of compounds **1**–**3**.

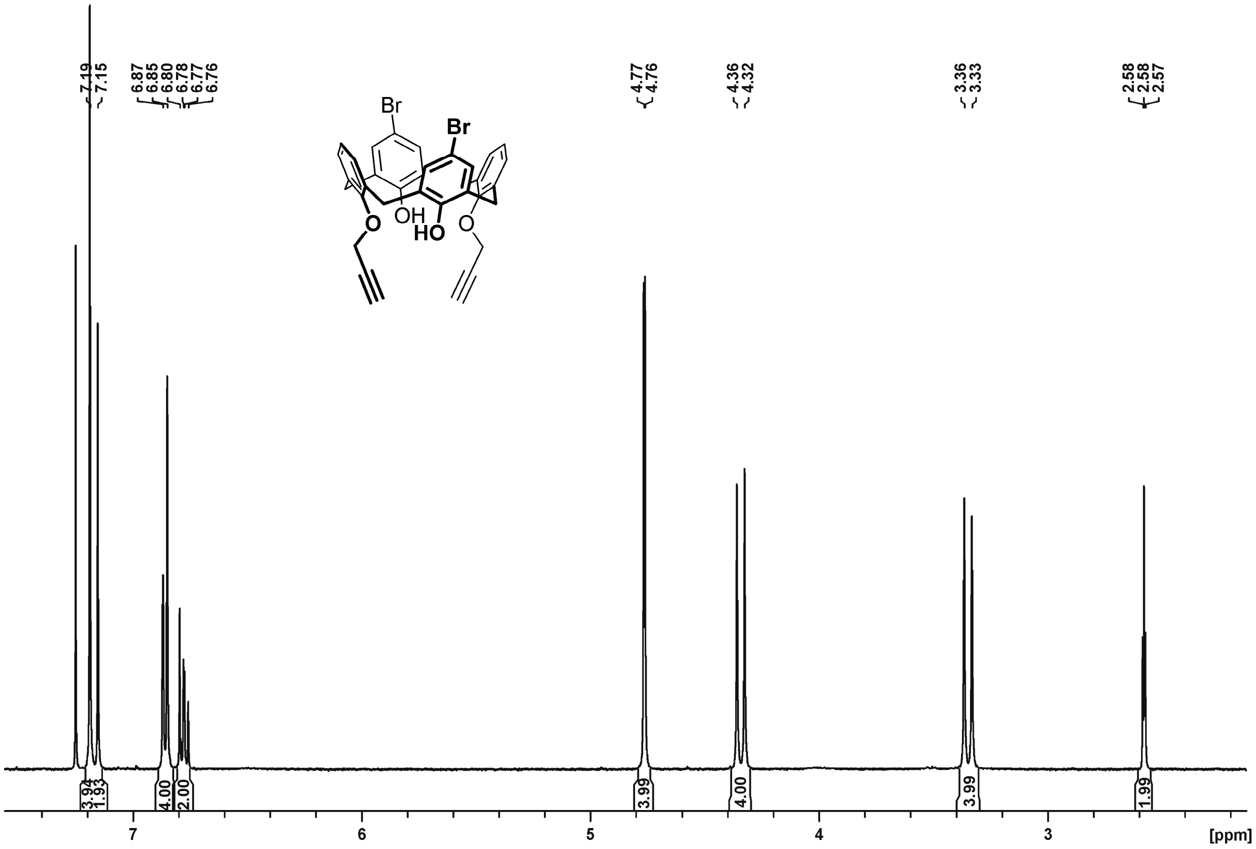

Figure S1. ^{1}H NMR spectrum of compound **1** (400 MHz, $CDCl_3$).

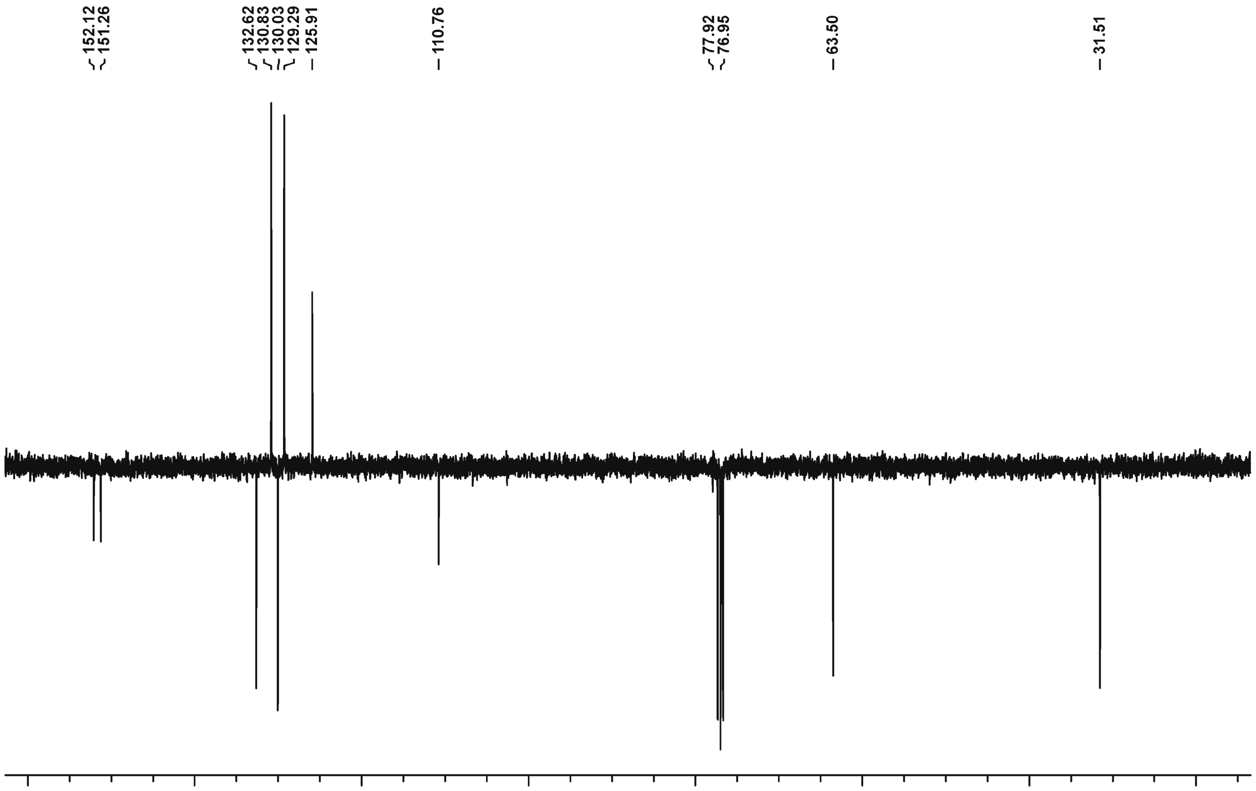

Figure S2. ^{13}C NMR spectrum (APT) of compound **1** (100 MHz, $CDCl_3$).

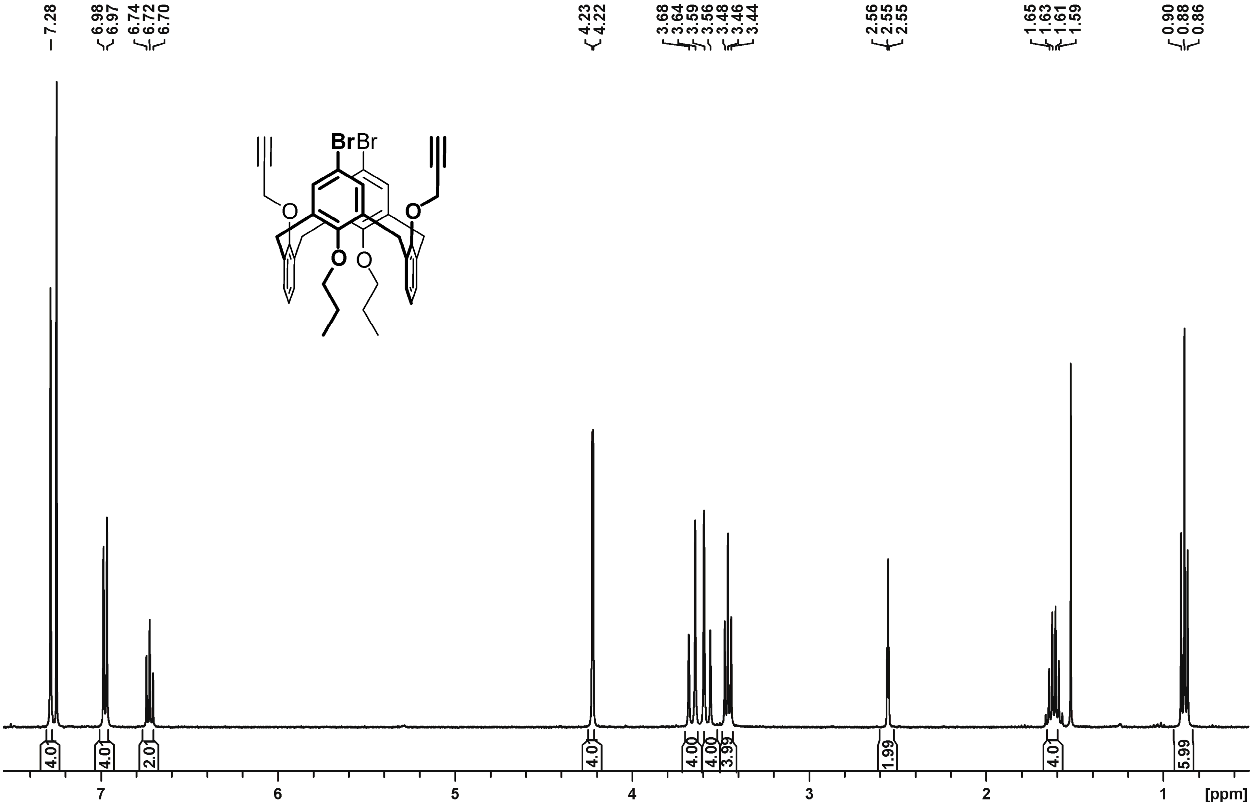

Figure S3. ¹H NMR spectrum of compound **2** (400 MHz, $CDCl_3$).

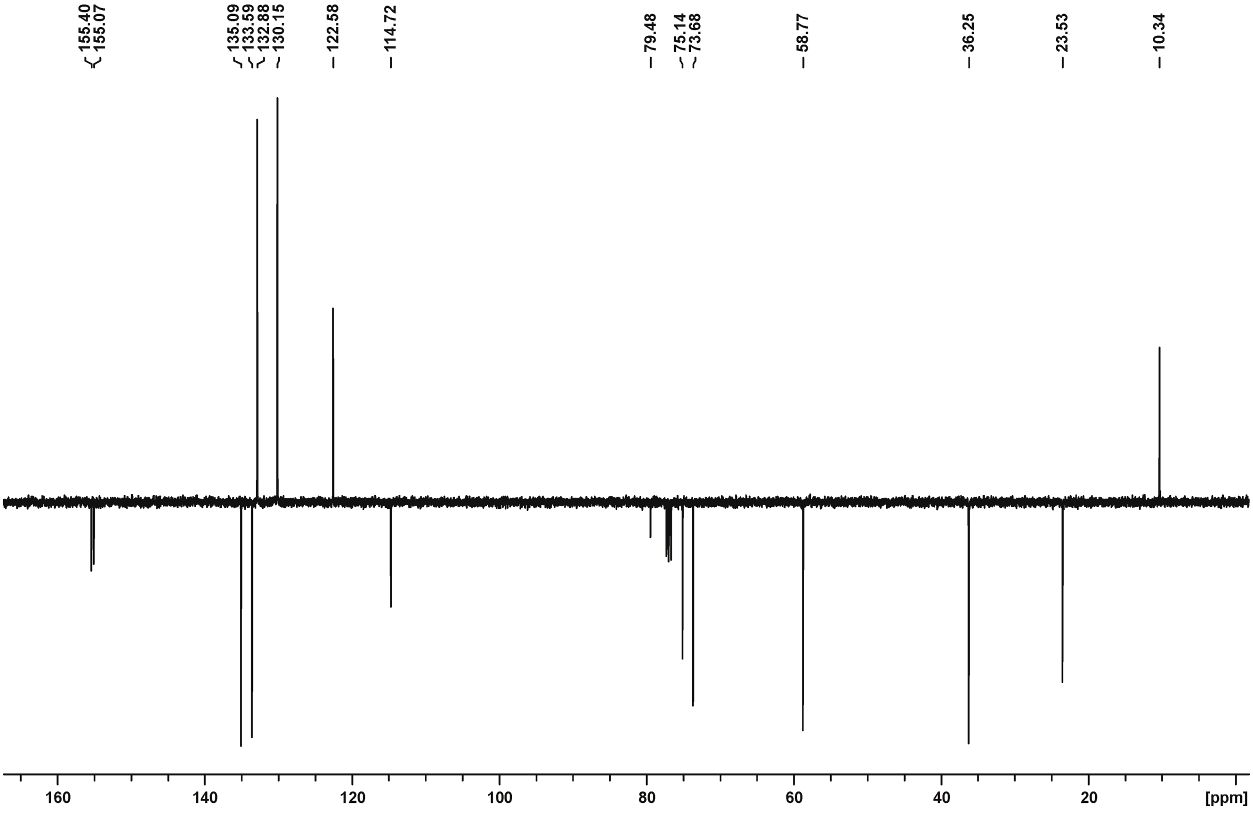

Figure S4. ¹³C NMR spectrum (APT) of compound **2** (100 MHz, $CDCl_3$).

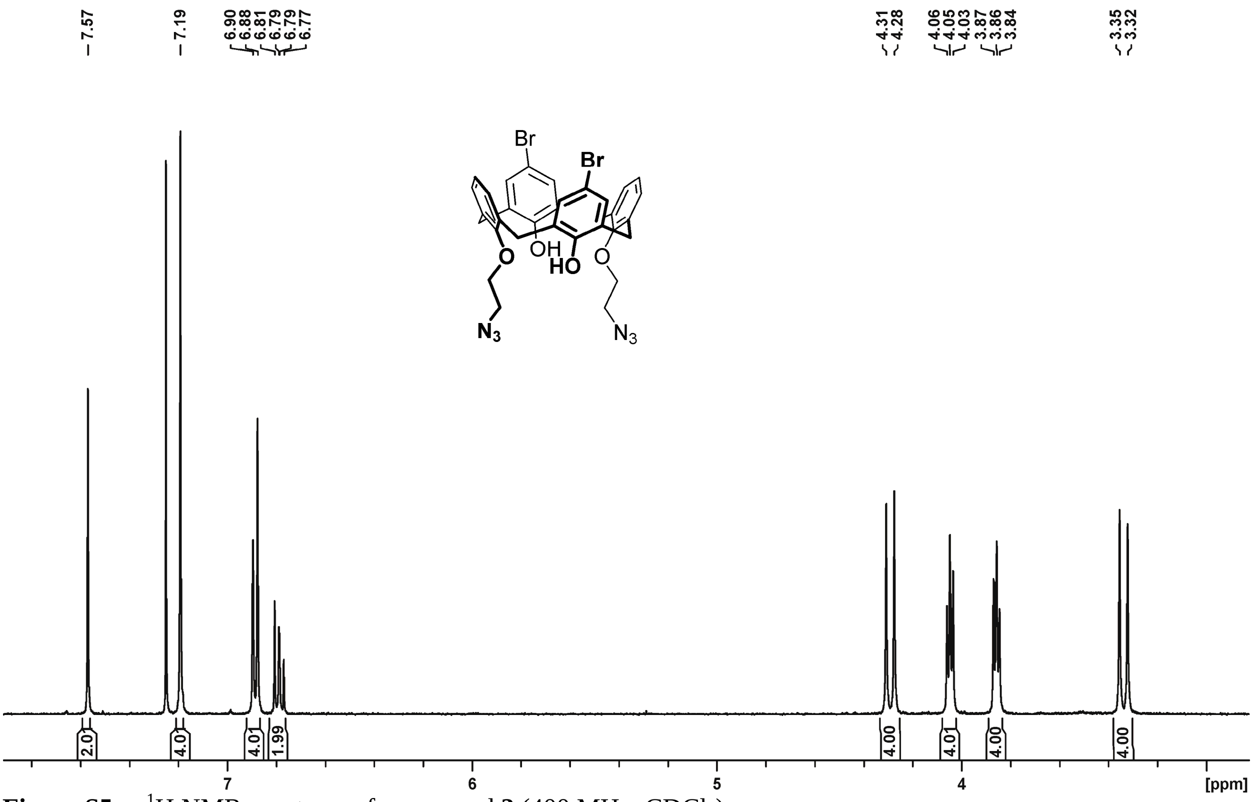

Figure S5. ^{1}H NMR spectrum of compound **3** (400 MHz, $CDCl_3$).

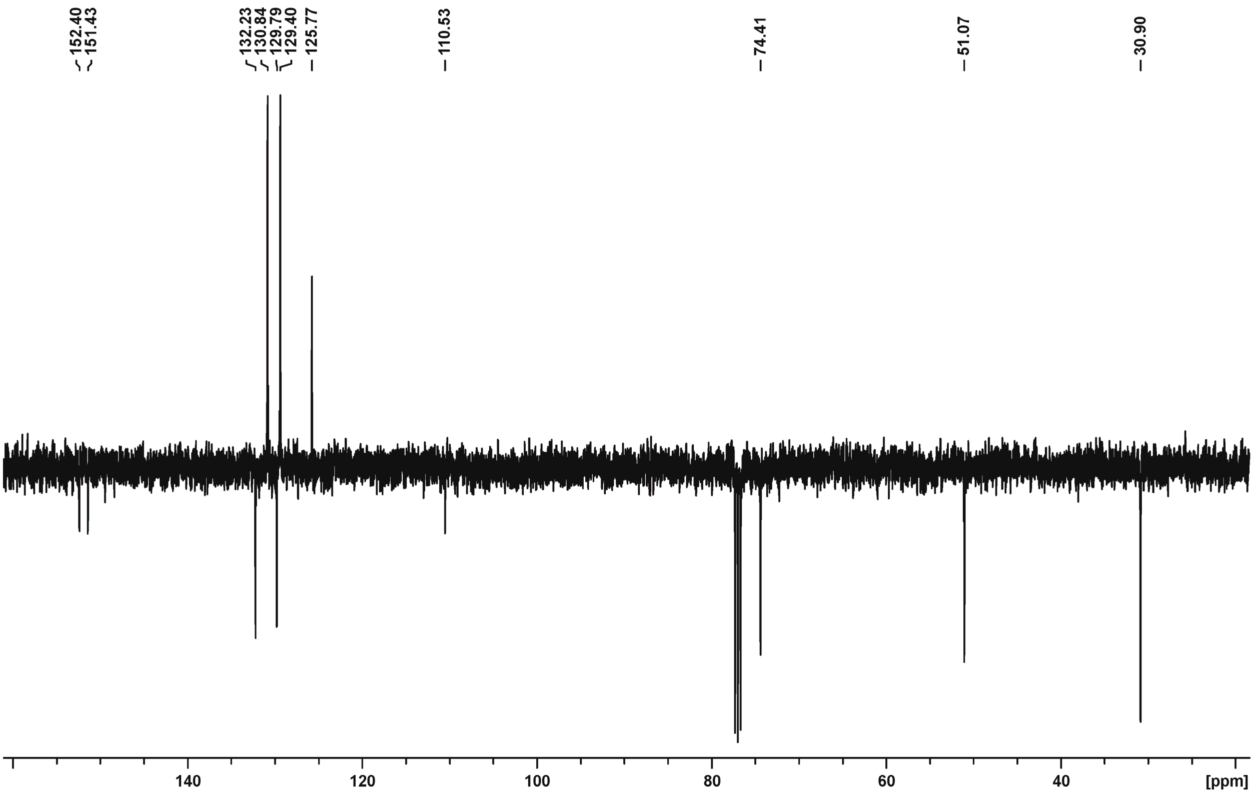

Figure S6. ^{13}C NMR spectrum (APT) of compound **3** (100 MHz, $CDCl_3$).
